# Mapping blood biochemistry by Raman spectroscopy at the cellular level†

**DOI:** 10.1039/d1sc05764b

**Published:** 2021-12-01

**Authors:** Victor V. Volkov, Jonathan McMaster, Joanna Aizenberg, Carole C. Perry

**Affiliations:** Interdisciplinary Biomedical Research Centre, School of Science and Technology, Nottingham Trent University. Clifton Lane Nottingham NG11 8NS UK Carole.perry@ntu.ac.uk +44 (0)115 8486695; School of Chemistry, Faculty of Science, The University of Nottingham Nottingham NG7 2RD UK; John A. Poulson School of Engineering and Applied Sciences, Harvard University 29 Oxford Street Cambridge MA 02138 USA

## Abstract

We report how Raman difference imaging provides insight on cellular biochemistry *in vivo* as a function of sub-cellular dimensions and the cellular environment. We show that this approach offers a sensitive diagnostic to address blood biochemistry at the cellular level. We examine Raman microscopic images of the distribution of the different hemoglobins in both healthy (discocyte) and unhealthy (echinocyte) blood cells and interpret these images using pre-calculated, accurate pre-resonant Raman tensors for scattering intensities specific to hemoglobins. These tensors are developed from theoretical calculations of models of the oxy, deoxy and met forms of heme benchmarked against the experimental visible spectra of the corresponding hemoglobins. The calculations also enable assignments of the electronic transitions responsible for the colour of blood: these are mainly ligand to metal charge transfer transitions.

## Abstract 2

We assign the electronic transitions responsible for the colour of blood and present a Raman imaging diagnostic approach for individual blood cells.

## Graphical abstract

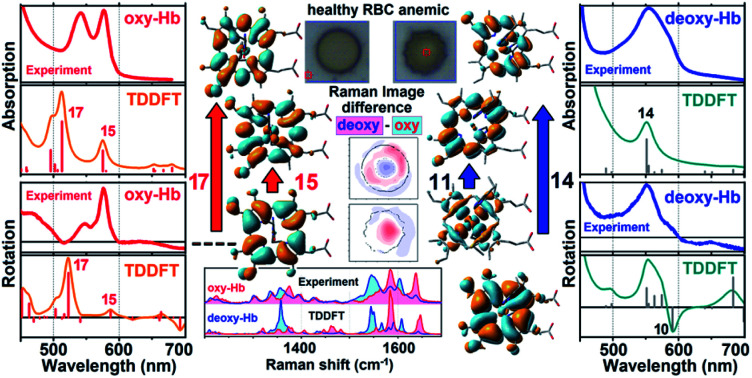

## Introduction

Porphyrins initially hosted by proteins for protection from light,^1^ became pivotal molecular components in the development of photosynthesis,^2,3^ respiration, and oxygen transport.^4,5^ For the latter, coordination of oxygen with heme (porphyrin + iron) plays a central role. In vertebrates, the supply of oxygen to tissues correlates with the colour of blood circulating in the vascular system. There has been interest in describing the colour of blood: for example, in 1674 John Mayow reported “when venous blood is placed in a vessel, the upper surface which is exposed to the air acquires a scarlet florid colour, although the blood at the bottom of the vessel appears as a dark purple; and yet it too if exposed to the air will after a short time become ruddy”.^6^ Thus, unsurprisingly present-day preventive medicine and intensive care both rely on monitoring the optical properties of blood to report on health.^7–11^

It is generally accepted that the optical absorption behaviour of blood in the visible spectral region (450 to 700 nm) should relate to the oxidation state of the Fe centre found in the centre of the protoporphyrin ring. Why is this important? Since the mid-1800s,^12^ the correlation of physical structure and electronic distributions has been fundamental to the understanding of molecular properties. This approach motivated the development of synthetic chemistry, experimental X-ray diffraction, optical spectroscopy, and methods of quantum chemistry.^13^ Early discussions on the nature of the heme, however, concerned mainly the geometric and electronic properties of the Fe–O_2_ moiety in oxy-hemoglobin (Hb).^14–16^ The numerous disputes and consequent further studies gave rise to the “ozone” model, which describe iron(ii) as coordinated to oxygen with an overall spin state of one.^17^ In the same time frame, experimental optical spectroscopy studies in the visible spectral range described the spectral response of blood with contributions of the three main forms of hemoglobin: oxy-Hb, deoxy-Hb and met-Hb.^18,19^ Despite efforts made to provide empirical assignments of the optical transitions for ferrimyoglobin,^20^ application of symmetry-adapted-cluster expansion theory for oxy-heme,^21^ and extended Huckel theory for porphyrins,^22^ there is still no systematic description of the optical electronic transitions of the main hemoglobins that determine the colour of blood.

To answer the challenge, in this contribution, first, using density functional theory^13,23–25^ for the central structural element specific to met-Hb, deoxy-Hb and oxy-Hb we reproduce their optical absorption and circular dichroism behaviour, as observed experimentally in the visible spectral range. Second, motivated by the Koopmans' theorem,^26^ we transform the transition density matrix^27^ and express the “lower” and the “upper” orbital components involved in the computed optical electronic transitions. Pairs of such orbital components constitute so-called natural transition orbitals (NTOs).^28^ By visualizing NTOs of the electronic transitions responsible for optical density in the visible spectral range we provide a systematic, up to the level of the theory used, description of their nature. At the practical level, an adequate description of pertinent electronic transitions is mandatory for quantitative Raman studies.^29,30^ Using the computed electronic transitions, we extract non-resonant and resonant Raman tensors of the heme systems and compare with the experimental Raman responses of the corresponding hemoglobins. The results enable us to analyse microscopy images of single red blood cells sampled at different Raman frequencies. The unique sensitivity of resonant Raman to heme states permits us to probe distributions of different hemoglobin forms in dependence on cellular physiology. We further discuss the potential development of quantitative Raman microscopy imaging approaches to advance the science of haematology.

## Methods

Human met-Hb in the form of lyophilized powder is obtained from Sigma-Aldrich, CAS Number: 9008-02-0. Using sodium dithionite, we convert met-Hb in solution into deoxy-Hb.^19^ We obtain oxy-Hb upon bubbling oxygen–air mixture through the solution of deoxy-Hb. Optical absorption of the prepared hemoglobins in the visible spectral range is confirmed using a Varian Cary 50 UV-Visible Spectrometer, Agilent Technologies, Santa Clara CA, US. Circular dichroism studies are accomplished using a J-810 spectrometer, JASCO International Co. Ltd. Tokyo, Japan. Resonant Raman microscopy studies of single red blood cells (pin prick from one of the authors. At the time the experiment was conducted NTU did not have any research governance arrangements specific to self-experimentation with which this project would be expected to comply. Compliance was confirmed by the University governance chair and chair of the ethics committee) is performed using a DXR microscope station from Thermo Fisher Scientific, Madison, Wisconsin, equipped with 100× microscopy objective, and 530 nm excitation radiation of 1 mW. Spectral resolution is 2 cm^−1^ according to the instrumental limit of the microscope operated with a 25 micron confocal aperture. Raman microscopy studies are conducted using samples deposited on an unprotected gold mirror, PFSQ05-03-M03 Thorlabs Ltd., Ely, United Kingdom. Raman activities at different sites of the selected cells (both cell types were naturally occurring in the sample) are sampled with a spatial resolution of 1 micron in both directions of the imaging plane. Spectra sampled at different sites, *i*, were analyzed to extract amplitudes of Raman activities, *A*_*ω*,*i*_, at frequencies of interests, *ω*, which are used in reconstructions of Raman activity microscopy images (RAM). The reconstruction are conducted according to
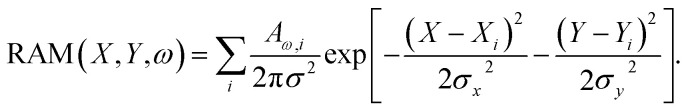
Here, we sum image projections of two-dimensional Gaussian source functions over all the defined sites *i*. Setting *σ*_*x*_^2^ = *σ*_*y*_^2^ = 0.5 μm^2^ provides the spatial full width of a source function. *X*_*i*_ and *Y*_*i*_ describe the position of the projection of the site *i* into the image plane. *X* and *Y* variables are sample distances from the site *i* in terms of the dimensions of detector pixels or displacements of a pinhole. Raman difference images are taken after normalizing the images (reconstructed for different wavelengths) by the sums of amplitudes at the sites *i*, in the image plane. As a result, the difference images describe spatial anti-correlation tendencies: intensity is zero at a site where the selected Raman activities contribute equally.

DFT structural optimizations, calculations of optical absorption, natural transition orbitals (NTO)^28^ pre-resonant Raman responses are conducted using the B3LYP functional^24^ within the Gaussian 09 program package.^31^ We use unrestricted UB3LYP functional^23^ to calculate the quintet spin state of the five-coordinate deoxy-form of the heme (with Fe^2+^), and the sextet state of the six-coordinated met-heme (with Fe^3+^) with the corresponding multiplicities 2·(4 × ½) + 1 = 5 and 2·(5 × ½) + 1 = 6. In the case of the oxy-heme structures, initially, we used restricted B3LYP functional. However, since a significant energy decrease (≈15 kcal mol^−1^) is confirmed upon the wavefunction stability test, further, an unrestricted UB3LYP functional is used to optimize and calculate properties of the bi-radical structure.^17^ We use 6-311++g(d,p) basis for hydrogen, carbon, nitrogen and oxygen atoms, while for iron ions we adopt the LANL2DZ basis set.^29^ Pre-resonant Raman spectra were computed using the cphf = rdfreq option by Gaussian at 532 nm, as we used in our experimental procedures.

For DFT optimizations, we prepare the initial structures of the Fe–porphyrin complex in the deoxy and oxy-forms with two (top and bottom) histidine ligands using X-ray based 2HHB^32^ and 1GZX^33^ entries as reported in the Structural Bioinformatics Protein Data Bank. To prepare the initial structure of the met-form of heme, we add a water molecule to coordinate with the iron ion in 2HHB.^32^ In the case of the oxy-form we considered both the geometry proposed by the X-ray study where the histidine proximal to the O_2_ ligand is doubly protonated^34^ and the Pauling geometry where the histidine proximal to the O_2_ ligand is singly protonated.^35^ Initial, structural optimizations are performed in vacuum. Then, since the dielectric constant at active sites in proteins may vary between 9 and 37,^36,37^ and, because the heme cavity provides mixed distant polar and nonpolar interactions, to approximate such an environment, we also perform optimization and subsequent analysis using the polarizable continuum model^38^ attuned to dimethyl sulfoxide, since it has an intermediate dielectric constant, and consists of non-bulky structural moieties of both polar and aprotic nature.

Since the basis set for the ring is augmented with diffuse functions and polarization but the selection for the iron is accounted for on a valence basis only, we may expect an offset in energies of electronic transitions, which may be large at the low frequency range. To explore this, we conduct single point calculations using the SDD: D95 basis set^39^ with Stuttgart/Dresden energy-consistent pseudo-potentials,^25^ and included a polarization function with a primitive f-electron set.

Since the structures are extracted from a protein environment, to understand instability in relative orientation of histidine in respect to the porphyrin plane, we conduct the initial DFT structural optimizations without any constraints. Angular constraints were added stepwise until the general relative orientation of the planes of the specific geometry, overall, was achieved. For the optimized structures we calculate an imaginary frequency mode smaller than −15 cm^−1^ specific to a rotation of histidine planes. When under constraint, a structure with very low imaginary frequencies, below 20 cm^−1^, may be considered as representative and equivalent to such at the stationary point.^40^

## Results and discussion

**Fig. 1:**
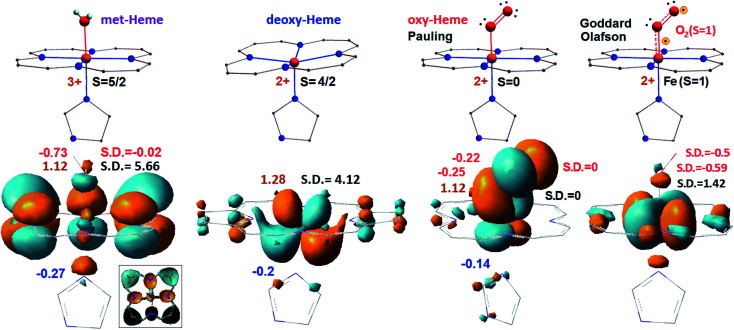
Electronic properties of the considered heme systems. At the top, graphical presentation of the central structural element specific to met-Hb, deoxy-Hb, oxy Hb in the geometry by Pauling^15^ and oxy-Hb under the configuration by Goddard and Olafson,^17^ from left to right. Formal charge and spin are at the left and right side of each structure, respectively. At the bottom, we show calculated NTO of the lower energy state of the first pairs calculated for the considered systems. For a better visibility of the d-electrons of the iron ion in the computed met-heme, we removed the π electron components of carbons and of nitrogen indicated as black in the boxed inset. Computed ESP atomic charges and spin densities are at the left and at the right side of each structure, respectively. We use red and blue for the numerical evaluations specific to oxygen and nitrogen atoms, as shown; we reserve brown and black to indicate values specific to iron atoms.

In Fig. 1, we compare formal charges and spin states of the iron ion for the optimized Fe-porphyrin systems (shown at the top) with atomic charges fitted to the electrostatic potential and calculated spin densities shown next to the natural transition orbitals (NTO) of the lower state of the first NTO pairs, see bottom, calculated using the UB3LYP functional with 6-311++g(d,p) basis for hydrogen, carbon, nitrogen and oxygen atoms, and LANL2DZ basis for the iron centre.^23,24^ We present data for met-Hb, deoxy-Hb, oxy-Hb in the geometry described by Pauling^15^ and oxy-Hb in the geometry and electronic configuration as considered by Goddard and Olafson.^17^

In all cases, the spin densities and atomic charges, computed for the two cases of oxy-heme, met-heme and deoxy-heme agree reasonably well with the formal assignments: for example, for deoxy-heme with formal assignment *S* = 4/2, DFT predicts spin density on iron to be 4.12. At the same time, the atomic charge of iron in met-heme is smaller than might be expected. Apparently, the ligand is dominant in providing the electron density for the metal centre: the nitrogen of the pyrrole ring A is almost neutral, while charges on the nitrogens of the pyrrole ring C and of the histidine are higher than might be expected: see Fig. 1 and ESI.† Based on the early discussion of the electronic properties of heme in hemoglobin,^14–17,35^ here, we compare the lower orbital components involved in the first (the lowest energy) optical transition. Specifically, if we now review the first NTO pairs, we note that there are considerable differences between electronic elements specific to met-Hb, deoxy-Hb, oxy-Hb (Pauling geometry) and oxy-Hb (configuration of Goddard and Olafson). For met-heme, the a_2u_ orbital component of the porphyrin ring dominates in the lower state of the first pair. The d_*xz*/*yz*_ component of the iron is prominent in deoxy-heme. For the Pauling geometry oxy-Hb the structure accounts π-type pairing of d_*yz*_ and 
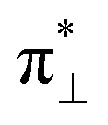
 orbital components in the lower state. The oxy form electronic components resemble orbitals of the oxy-heme as computed previously using both, CASSCF/MM and B3LYP/MM levels of theory.^35^ And last, for the bi-radical structure proposed by Goddard and Olafson, the d_*xy*_ orbital of the iron ion is involved in an antibonding interaction with histidine nitrogen atoms and dioxygen.

**Fig. 2:**
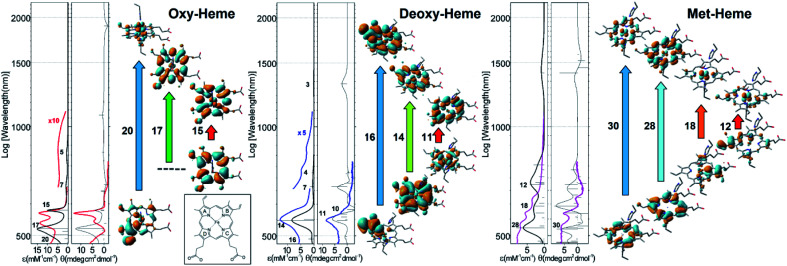
Optical spectroscopy in the visible spectral range and results of TD-DFT studies. Experimental (coloured lines) and theoretical (black lines) optical absorption and circular dichroism spectra for the main three forms of heme; NTO pairs that account for the main spectral features in the visible spectral range. Here for simplicity, we sum contributions of α and β electrons. The ESI† provide details and expanded views of the computed spectra of the considered heme systems using two level of theory as we describe. The boxed inset provides notation for the pyrrole rings of heme.

In Fig. 2 we compare experimental visible (VIS) absorption and circular dichroism (CD) spectra with theoretical predictions for the model systems and show the NTO orbital pairs responsible for the main spectral features. To anticipate possible limits of the theory level used, in the ESI† we also explore spectral responses in the visible spectral range for single point calculations using a higher level SDD: D95 basis set^24^ with Stuttgart/Dresden energy-consistent pseudo-potentials,^25^ including a polarization function with a primitive f-electron set. The calculated spectral responses in the visible spectral range using different theory levels demonstrate comparable amplitudes and frequencies of the main resonances.

According to our calculations for the bi-radical form of oxy-heme, we assign the main optical absorption bands and optical activities of oxy-hemoglobin at 576 and 541 nm to transitions which involve natural transition orbital (NTO) pairs 15, 17 and 20. The lower state of the 15^th^ and 17^th^ pairs contains a π-bonding orbital of the porphyrin ring. The upper state of the 15^th^ transition contains the π-type pairing of iron d_*yz*_ and oxygen 
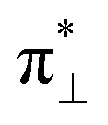
 orbital component. The upper state of the 17^th^ pair contains d_*xz*_-orbital of the iron in antibonding arrangement with respect to the anti-bonding π* orbital of the porphyrin ring. The lower state of the 20^th^ transition is of π bonding character and mainly localized on the pyrrole ring B (see inset in Fig. 2) and its vinyl group. The upper state of the 20^th^ NTO pair includes the d_*yz*_-orbital component of the iron ion, which is antibonding in respect to the π* component of the ligand oxygen. The ligand to metal charge transitions described here determine the “scarlet florid colour” of oxygenated blood in vertebrates; the d–d transitions dominate in the mid infrared and not in the visible region: the ESI† provides details and expanded views of the computed spectra of the considered heme systems using two levels of theory as we describe.

For deoxy-Hb, which is observed as the dark purple colour of blood in veins,^6^ our computations predict that NTO pair 14 may explain the dominant optical absorption of this species at 554 nm. According to the TD-DFT results, the optical absorption involves a charge-transfer transition from the bonding π orbital of porphyrin to the d_*xz*_-orbital of iron in antibonding configuration with the π* component of the porphyrin. Additionally, theory predicts that the low- and the high-energy side of the main band in the visible may be ascribed to the transitions expressed by the 11^th^ and 16^th^ NTO pairs, respectively: Fig. 2. Calculated optical activities specific to the NTO pairs 11, 14 and 16 are in good agreement with experimental CD. The lower-state of the 11^th^ pair is dominated by the d_*xy*_ electronic component of the iron ion in anti-bonding configuration with respect to the n-lone pairs of the porphyrin nitrogen atoms. The upper state of the 11^th^ NTO is a π* antibonding orbital on the porphyrin. The 16^th^ pair indicates a transition from a π bonding orbital localized at the vinyl moiety and the proximal pyrrole ring A to the π* bonding component of the vinyl moiety admixed with π* bonding delocalization over the whole porphyrin. Here, it is important to note that application of extended Huckel theory suggested a_1u_(π) → e_g_(dπ) charge transfer at 1.8 eV (689 nm) for Fe^2+^ high spin symmetric porphyrins.^22^ Here, the 14^th^ NTO contains a contribution of such a transition.

Description of electronic properties of met-Hb in the visible spectral range is challenging due to the larger number and complexity of optical transitions in this spectral region. The very broad optical absorption suggests that the porphyrin in met-Hb may resemble the family of paleo-chromophores used by early evolutionary life forms for photo-protective screening against UV-VIS radiation at Earth surface under anaerobic conditions.^1^ Nonetheless, TD-DFT suggests that the quartet of experimentally observed transitions at 632, 576, 536 and 500 nm may be assigned to NTO pairs 12, 18, 28 and 30, respectively, as calculated for this heme system, despite a frequency mismatch tendency: with the calculated lower frequency transitions being red shifted compared to the experimental data. An improvement of frequency matching can be obtained using a larger basis set for the iron ion: see the ESI.† Calculated optical activities are in a general agreement with the experimental data, save the frequency mismatch observed for the lower energy transitions. In describing the nature of the anticipated optical transitions, we show that the lower states for pairs 12 and 18 involve π bonding orbitals localized on pyrrole rings A and C, respectively, while other lower states are π bonding orbitals that involve the whole porphyrin ring. All upper states of the transitions contain the d_*yz*_ electronic component of the iron ion. This component is identical and dominant in the upper orbitals for the 12^th^ and the 18^th^ pair. For the 28^th^ and 30^th^ pairs, the d_*yz*_ orbital component finds non-bonding coordination with the prominent π* bonding and π* antibonding orbitals of the porphyrin ring in the upper state of the 28 and 30^th^ pairs, respectively. In the ESI,† we provide more detail on the optical transitions in the visible spectral range as calculated for met-heme using different basis sets. The direct outcome of our computational studies supported by experimental observations is that we are able to provide a consistent, up to the level of the theory, description of the nature of the electronic transitions that determine the colour of blood in vertebrates including humans.

Understanding of molecular properties critically depends on correlation of structure and electronic properties. For example, an adequate description of relevant electronic transitions is necessary for quantitative Raman analysis. Frequency-resolved Raman spectroscopy of red blood cells has emerged as a promising approach to provide systematic insight into the biochemistry of blood at the sub-cellular level.^41,42^ Nonetheless, the application of resonant^41^ and non-resonant^42^ approaches to the quantitative analysis of blood remains challenging. Specifically, pre-resonant spectral responses are specific to the excitation wavelength,^43^ which is specified for the computations. Specifying the excitation wavelength at 532 nm, we define frequency detunings in respect of all electronic resonances as computed by TDDFT. The amplitudes of resonances from different hemoglobins are expected to reflect incomplete orientation distributions due to the architecture of cytosol and membrane associated biochemistry,^39,40^ and rapid molecular transport and reshaping of erythrocyte cell envelopes are stimulated under cell fragmentation, oxidative injury, immune-mediated damage, congenital abnormalities and other anaemia tendencies.^7^ It is important to note that variances in incomplete orientation averaging, transport and reshaping restrict application of unitary transformation techniques^44^ for quantitative identification of distributions of biomarkers in space.

**Fig. 3:**
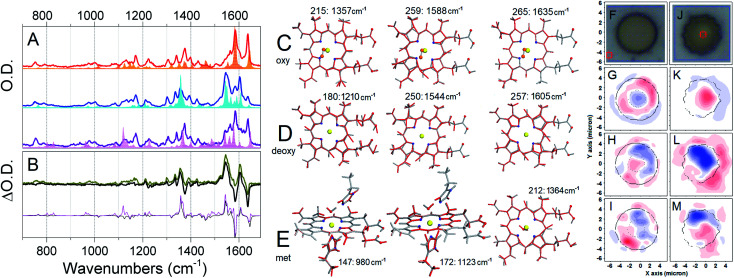
Raman spectroscopy and microscopy studies. (A) Pre-resonant experimental Raman spectra of oxy (thick red line), deoxy (thick blue line), and met (thick purple line) hemoglobin compared with calculated pre-resonance Raman spectra (filled profiles) calculated for the equivalent forms of the Fe–porphyrin complex: description of the normal modes is in the ESI.† (B) Raman difference spectra (response of deoxy-Hb is subtracted from that of oxy-Hb) using experimentally detected (thick black line) and calculated (thin black line) responses. Thick yellow and thin magenta lines account for the addition of experimentally detected and calculated contributions (5%) specific to the met-form of the heme. Structural changes (red lines) along the normal modes (selected for Raman microscopy image reconstructions) specific to oxy (C), deoxy (D), and met (E) hemoglobin. To help visualization, we remove histidines in panels (C) and (D). Bright field (F) and Raman difference images IM_deoxy_–IM_oxy_ (G), IM_oxy_–IM_met_ (H), and IM_deoxy_–IM_met_ (I) for a healthy discocyte using Raman signals experimentally detected and reconstructed at 1364, 1605 and 1636 cm^−1^, which we assigned to the met, deoxy, and oxy forms of hemoglobin, respectively: see panels (A)–(E). Bright field (J) and Raman difference images IM_deoxy_–IM_oxy_ (K), IM_oxy_–IM_met_ (L), and IM_deoxy_–IM_met_ (M) of an echinocyte cell using the same Raman signals as in panels (F)–(I). Microscopy studies were conducted on red blood cells deposited on dry gold-coated glass substrates.

To approach the study individual red blood cell biochemistry using Raman microscopy, first, we depart from empirical assignments^45^ and describe pertinent vibrations as normal modes according to our computational results. As an example, Fig. 3A shows pre-resonant Raman spectra measured for the three hemoglobins together with that calculated for the considered heme systems using an excitation wavelength of 532 nm. Also, in Fig. 3B, we present pre-resonant Raman difference spectra where the spectrum of the deoxy-form is subtracted from the spectrum of the oxy-form alone and with contribution of the met-form using experimental dispersions specific to the three hemoglobins and theoretical responses for the three heme forms. The experimental difference spectra in Fig. 3B are in general consistent with reported Raman micro-spectroscopy on the tense-relaxed state transition of hemoglobin.^41^ Comparing the experimental and the theoretical Raman resonances, as presented in Fig. 3A and B, we assign resonances at 747, 991, 1364 and 1541 cm^−1^ to met-Hb; the Raman activities at 1377, 1399, 1588 and 1636 cm^−1^ to oxy-Hb; and the signals at 1545 and 1605 cm^−1^ to deoxy-Hb: a detailed review is provided in the ESI.†

Next, we sample Raman responses of two different blood cell types, namely discocyte (healthy) and echinocyte (abnormal) cells (Fig. 3F and J), reconstruct images at the selected Raman resonances, and normalize on the sums of its intensities for each cell system. Further, according to the theory-based assignment of resonances, and the results of Raman experimental studies on hemoglobins in solution (Fig. 3A and B), we group the images into three sets characteristic of the three hemoglobins and take difference images according to the three types: IM_deoxy_-IM_oxy_, IM_oxy_-IM_met_, and IM_deoxy_-IM_met_. In Fig. 3G–I and K–M we present selected difference images for representative discocyte and echinocyte cells, respectively at the selected representative frequencies. ESI† provide an extended library of difference images consistent with the assignments of resonances. Taking differences between normalized images we express anti-correlation tendencies in spatial distribution of the three distinct hemoglobins: the difference intensity is zero at a site where Raman activities of a selected pair contribute equally. Blue coloured regions indicate where the species, the Raman response of which we subtract, dominates, and *vice versa* for red coloured regions. Therefore, Fig. 3G–I and K–M compare correlations and anti-correlations for the spatial distribution of met, deoxy and oxy hemoglobins in healthy (discocyte) and leaky (echinocyte) red blood cells.

As an example, Fig. 3G demonstrates that in a healthy discocyte there is a slight tendency for deoxy-Hb to be displaced from the central region and from the sides of the cell (where the oxy-form tends to be more abundant) toward the centre of the toroidal channel. In contrast, Fig. 3K indicates that in the echinocyte the distribution is markedly altered with the deoxy-Hb tending to occupy the central region. This suggests a major disruption of the internal distribution of the two main hemoglobins (oxy and deoxy). The result is interesting to correlate with the reported height increase in the centre of echinocytes as compared to discocytes.^46^ Furthermore, the Raman difference image suggests that redistribution of oxy and deoxy-Hb in the echinocyte is associated with cytoskeletal rearrangements that form structures known as spicules, which are characteristic of echinocytes.^46^Fig. 3K–M show that regions of higher red/blue intensities tend to locate between spicules. Therefore, we may consider that spicules are involved in leaking of hemoglobins outside the cell: indeed, several hemolytic anemias have been reported in association with echinocytosis.^47^ The observed correlations have the potential to open up a new methodological perspective in biomedical diagnostics of red blood cell pathologies and stress reaction mechanisms.

In Fig. 3H and L we explore if the distribution of met-Hb anti-correlates in space in respect to the oxy form of the protein. The blue ring in Fig. 3H spatially correlates with the red ring in Fig. 3G: the blue ring pattern being slightly shifted towards the centre. This suggests that the “loss” of the deoxy-Hb (due to conversion into met-Hb) is on-going and this is more effective in the internal segment of the toroidal channel. This “loss” is more obvious in the echinocyte where the intense blue signature in the central region in Fig. 3L, correlates with the red signature in the central region in Fig. 3K. Furthermore, if we compare the red regions in Fig. 3H, with the blue pattern in Fig. 3G, we show that there are higher levels of the oxy-form (red regions in Fig. 3H) in the areas where oxy and deoxy forms of the protein are equally present in Fig. 3G. This is the region in Fig. 3G, where the difference signal is zero – along the silhouette line on the inner side. At the same time, it is clear that the oxy form dominates in the leaky content outside the echinocyte: as observed in the red pattern in Fig. 3L. The dominance of the oxy form there is most likely due to exposure of the cell to air during spectral analysis.

In Fig. 3I and M we explore if distribution of met-Hb anti-correlates in space with respect to the deoxy-form of the protein. Here it is important to note that preserving hemoglobin either in oxy or in deoxy-form is critically important for the main function of erythrocytes. In this pair, the reduced form is conserved in the presence of 2,3-bisphosphoglycerate.^48^ Nonetheless, some loss due to conversion of the deoxy-form into met-Hb does happen. Once sorted by evolution for photo-protective screening against UV-VIS radiation as expected at Earth surface with anaerobic atmosphere,^1^ the paleo-form of heme in met-Hb is a burden in respiration. In fact, it is constantly amended by cytochrome-b_5_–MHb reductase.^48^ Therefore, Fig. 3I and M could be considered to report on the on-going activity of the enzyme. Specifically, the blue regions in Fig. 3I and M indicate where cytochrome-b_5_–MHb reductase is slow or failing in its “duty”. Results in Fig. 3I indicate that cytochrome-b_5_–MHb reductase is effective in the centre of the toroidal channel of the discocyte. It is interesting that the enzyme appears to be active in the centre of the echinocyte but fails to prevent accumulation of the met-form in the region of the external spicules. Overall, the results of our Raman microscopy studies indicate that the shape of blood discocyte cells is important to control met-Hb.

We note here that the response of the same hemoglobin in diverse difference maps may provide information on local orientational variances. For example, the bright red signature (possibly, of a local cluster of the deoxy form) at the cell boundary in Fig. 3I. We do not observe consistent Raman scattering in Fig. 3G. This may be a signature of oxidative stress, under which it is reported that different forms of hemoglobin may associate with membrane band 3 complexes. The role of variances in distribution of different forms of hemoglobins next to the membrane and implications for inter-conversion rate between the forms is discussed in the literature.^39,40^ Association with a membrane would imply local incomplete orientational averaging. Since in the present studies we extract libraries of Raman tensors (both resonant and non-resonant) it is possible to model local anisotropies according to the procedures as we discussed recently.^30^ This would be helpful to detail cellular mechanisms of blood oxidative stress,^10,11^ methemoglobinemia^49,50^ and other anaemic tendencies.^7,8,51^ Due to the unique specificity of Raman to the state of heme, the approach described herein offers a sensitive diagnostic to tackle blood biochemistry at the cellular level *via* monitoring of redistributions of the main forms of hemoglobin, which may not be achieved easily using any other microscopy technique. Nonetheless, to take our approach forward into intensive care would require developing statistical assessments using fast imaging of cells at specific Raman resonances under physiological conditions exploiting microfluidic devices. Furthermore, combined with confocal fluorescence microscopy, the approach may help to correlate redistributions of hemoglobins with cell cytoskeleton dynamics and ionic signaling. Along this line, testing red blood cell structural reactivity, in response to external biophysical and biochemical interventions, may assist development of benchmark protocols to support developments in Haematology.

## Funding

Dr Victor V. Volkov and Dr Carole C. Perry gratefully acknowledge funding from AFOSR FA9550-16-1-0213 and FA9550-20-1-0206.

## Data availability

All data needed to evaluate the conclusions in the paper (UV-VIS spectra, electronic properties of all the considered structures and normal modes descriptions) are present in the paper and/or the ESI.† Additional data related to this paper may be requested from the author Carole.Perry@ntu.ac.uk on reasonable request.

## Author contributions

All authors contributed to the preparation of the manuscript. V. V. V. and C. C. P. conducted theoretical studies with participation of J. A. Raman studies were performed by V. V. V. Optical electronic spectroscopy was accomplished by V. V. V., J. M., and C. C. P. The final version of the manuscript has been approved by all authors.

## Conflicts of interest

The authors declare no competing interests.

## Supplementary Material

SC-013-D1SC05764B-s001
