# Supplementary material for: Mapping blood biochemistry by Raman spectroscopy at the cellular level
Source: Chem Sci. 2021 Dec 1;13(1):133–40. doi: 10.1039/d1sc05764b (PMC8694331; doi:10.1039/d1sc05764b)
Supplement: SC-013-D1SC05764B-s001 [file SC-013-D1SC05764B-s001.pdf]

Electronic Supplementary Material (ESI) for Chemical Science.
This journal is © The Royal Society of Chemistry 2021

Supplementary Materials for

Mapping blood biochemistry by Raman Spectroscopy at the cellular level

Victor V. Volkov, Jonathan McMaster, Joanna Aizenberg, Carole C. Perry.

Correspondence to: carole.perry@ntu.ac.uk

This PDF file includes:

Supplementary Text
Figs. S1 to S33

ELECTRONIC PROPERTIES OF DEOXY-HB

Here, we compare experimental optical absorption (left) and circular dichroism (right) spectra of deoxy-Hb with calculated spectra for deoxy-heme we extracted from 2HHB entry as reported in the Structural Bioinformatics Protein Data Bank using the two indicated theory levels. Using UB3LYP: 6-311++g(d,p)/LANL2DZ we optimize structure and calculate optical absorption and circular dichroism. Then, we repeat calculations of optical properties but using SDD:D95 theory for the structures optimized with UB3LYP: 6-311++g(d,p)/LANL2DZ. The main purpose of application of SDD:D95 is to see if this high level theory would provide confirmation that the dominant resonances identified using the lower level of theory would not shift significantly. From the data presented it can be seen that both theories provide comparable optical absorption spectra. Circular dichroism calculated using UB3LYP: 6-311++g(d,p)/LANL2DZ resembles the experimental result reasonably well save the amplitudes of the 7 and 10th resonances are slightly overestimated. These could be accounted for if we would adopt spectrally broader line-shapes for these resonances upon spectral convolution. In Fig. S1 we show spectral dispersions using the same line-shapes, though for the same frequency fluctuation dynamics, a line-shape in the lower frequency range is expected to be broader. Amplitudes of circular dichroism transitions using SDD:D95 do not resemble the experimental.

Here, we see that changing the level of theory for the structure optimized under UB3LYP: 6-311++g(d,p)/LANL2DZ does not help to predict the relative components of electric and magnetic transitions. The contribution of the NTO pair 14 may be assigned to describe the colour of deoxy hemoglobin in blood. Here it is important to stress that d-d transitions of the iron do not play a significant role in the transition. In Fig. S2 we present images of the selected NTO pairs for this structural case. We can clearly see that ligand electrons and a small contribution of ligand-to-metal charge transfer of resonance 14 determines the colour of blood in veins. Further, we list the main localized α and β electronic contributions in the imaged pairs. In a case α and β contribute into a pair, the image includes both contributions.

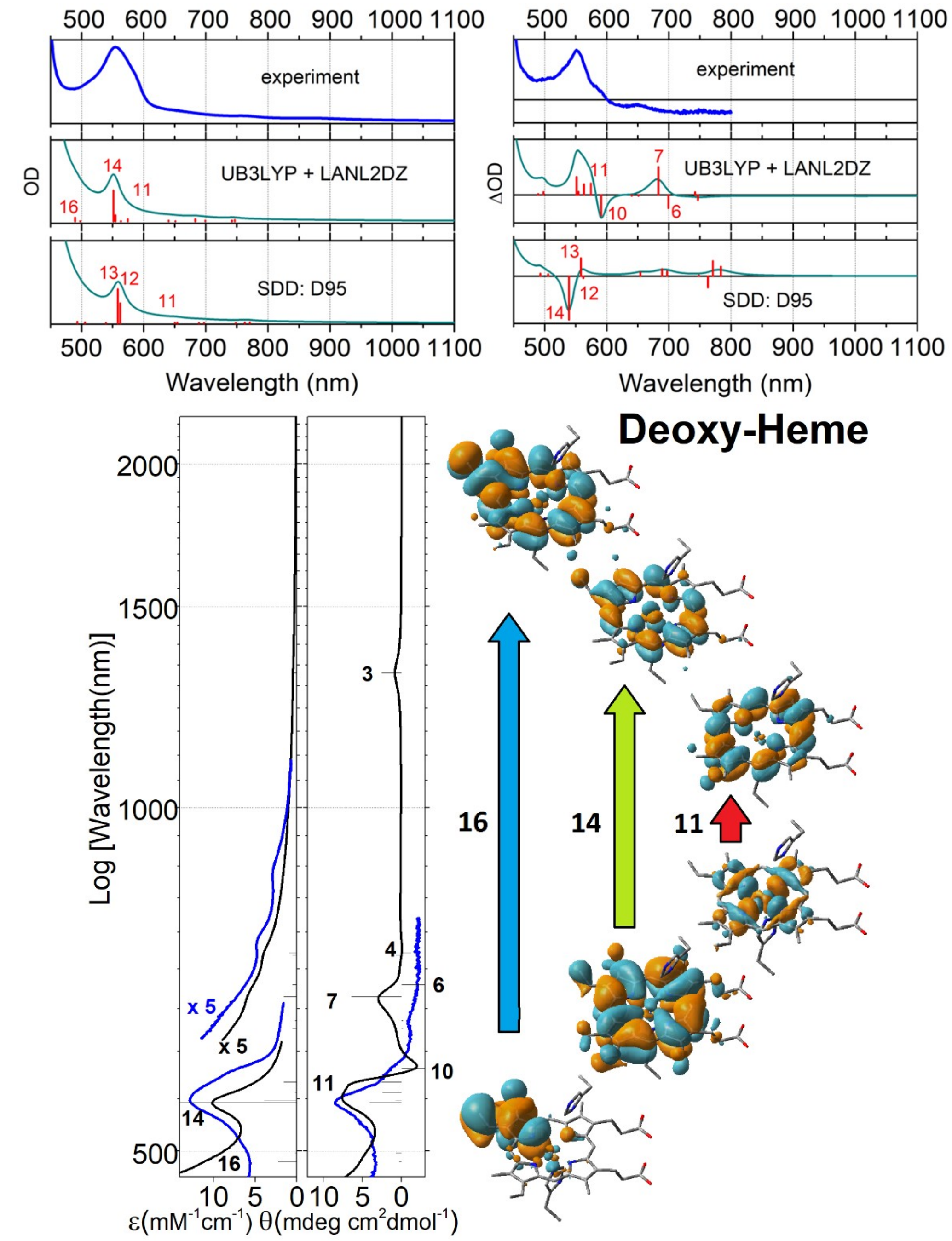

Fig. S1. Optical spectroscopy in the visible spectral range and results of TD-DFT studies for deoxy-Hb. Upper panels: optical absorption (left) and circular dichroism (right) spectra experimental and calculated for deoxy-heme we extracted from 2HHB entry as reported on the Structural Bioinformatics Protein Data Bank using UB3LYP: 6-311++g(d,p)/LANL2DZ and SDD:D95 theory level. Numbers indicate NTO pairs. **Lower panels:** Experimental (coloured lines) and theoretical (black lines) optical absorption and circular dichroism spectra for the system at SDD:D95 theory; NTO pairs that account for the main spectral features in the visible spectral range. Here for simplicity, we sum contributions of α and β electrons.

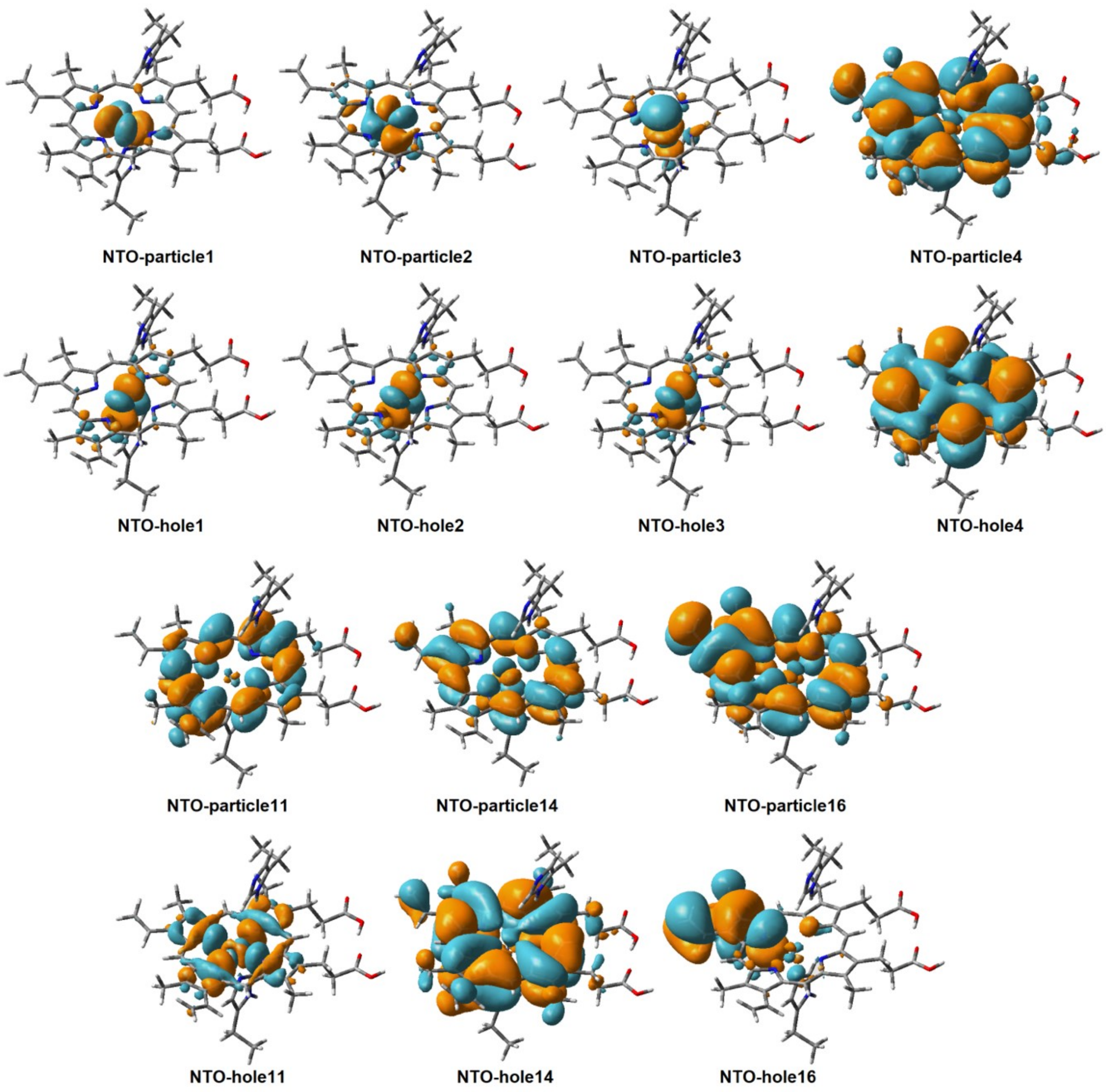

Fig. S2.

Electronic properties of deoxy-heme. NTO pairs for deoxy-heme using UB3LYP: 6-311++g(d,p)/LANL2DZ. Pairs 1-4 contribute to optical absorption at 6805, 5381, 1312 and 747 nm. Pairs 11, 14 and 16 contribute to resonances at 575, 551 and 489 nm that determine optical absorption in the visible spectral range. In a case where α and β electrons participate in the optical transition, we sum their contributions properly scaled for simplicity in visual content. However, in the text, below, we quantify the contributions of α and β electrons.

NTO pair 1: main localized contributions
Beta occ 206 OE=2.579 is Fe6-d=0.91
Beta vir 207 OE=2.579 is Fe6-d=0.95
NTO pair 2: main localized contributions
Beta occ 206 OE=2.462 is Fe6-d=0.91
Beta vir 207 OE=2.462 is Fe6-d=0.92

NTO pair 3: main localized contributions
Beta occ 206 OE=2.096 is Fe6-d=0.92
Beta vir 207 OE=2.096 is Fe6-d=0.87

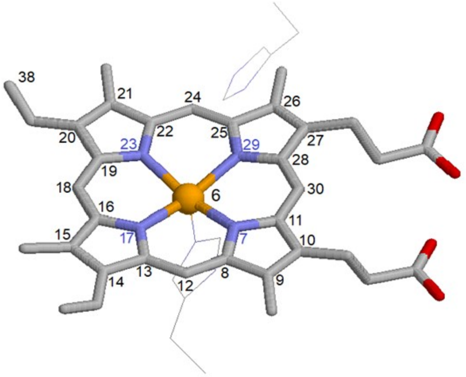

NTO pair 4: main localized contributions
Alpha occ 209 OE=0.344 is C19-p=0.09
Alpha occ 210 OE=0.656 is C18-p=0.15 C30-p=0.13 C24-p=0.11 C12-p=0.11
Alpha vir 211 OE=0.656 is C18-p=0.15 C30-p=0.13
Alpha vir 212 OE=0.344 is C12-p=0.13 C24-p=0.12
Beta occ 205 OE=0.312 is C19-p=0.08
Beta occ 206 OE=0.686 is C18-p=0.12
Beta vir 207 OE=0.686: C18-p=0.13 C30-p=0.11
Beta vir 208 OE=0.312: C12-p=0.11 C24-p=0.11

NTO pair 11: main localized contributions
Alpha occ 210 OE=1.893: Fe6-d=0.36 N17-p=0.10
Alpha vir 211 OE=1.893: C18-p=0.08

NTO pair 14: main localized contributions
Alpha occ 209 OE=0.278: C12-p=0.13 C18-p=0.13 C30-p=0.12 C24-p=0.12 N23-p=0.11
Alpha occ 210 OE=0.734: C22-p=0.09
Alpha vir 211 OE=0.734: C18-p=0.08
Alpha vir 212 OE=0.278: C12-p=0.07
Beta occ 205 OE=0.388: C22-p=0.09
Beta occ 206 OE=0.593: C12-p=0.15 C24-p=0.15 C30-p=0.15 C18-p=0.14
Beta vir 207 OE=0.593: C18-p=0.07
Beta vir 208 OE=0.388: Fe6-d=0.08

NTO pair 16: main localized contributions
Alpha occ 210 OE=1.088: C21-p=0.26 C38-p=0.19 C20-p=0.19 N23-p=0.19
Alpha vir 211 OE=1.088: C21-p=0.15 C38-p=0.13 C20-p=0.11
Beta occ 206 OE=0.805: C21-p=0.27 C38-p=0.21 C20-p=0.19 N23-p=0.17
Beta vir 207 OE=0.805: C21-p=0.16 C38-p=0.15 C20-p=0.12

ELECTRONIC PROPERTIES OF OXY-HB

Here, we compare experimental optical absorption (left) and circular dichroism (right) spectra of oxy-Hb with calculated spectra for oxy-heme structure we extracted from 1GZX entry as reported in the Structural Bioinformatics Protein Data Bank using the indicated theory levels. Using UB3LYP: 6-311++g(d,p)/LANL2DZ we optimize structures of the singlet and of the biradical, and calculate optical absorption and circular dichroism spectra. Here, finding a stable wavefunction for the biradical provides additional energy optimization of 15.9 kcal/mol. Then, we repeat calculations of optical properties but using SDD:D95 level of theory for the structures optimized with UB3LYP: 6-311++g(d,p)/LANL2DZ. The main purpose of application of SDD:D95 is to see if this higher level theory would confirm that the dominant resonances would not shift significantly.

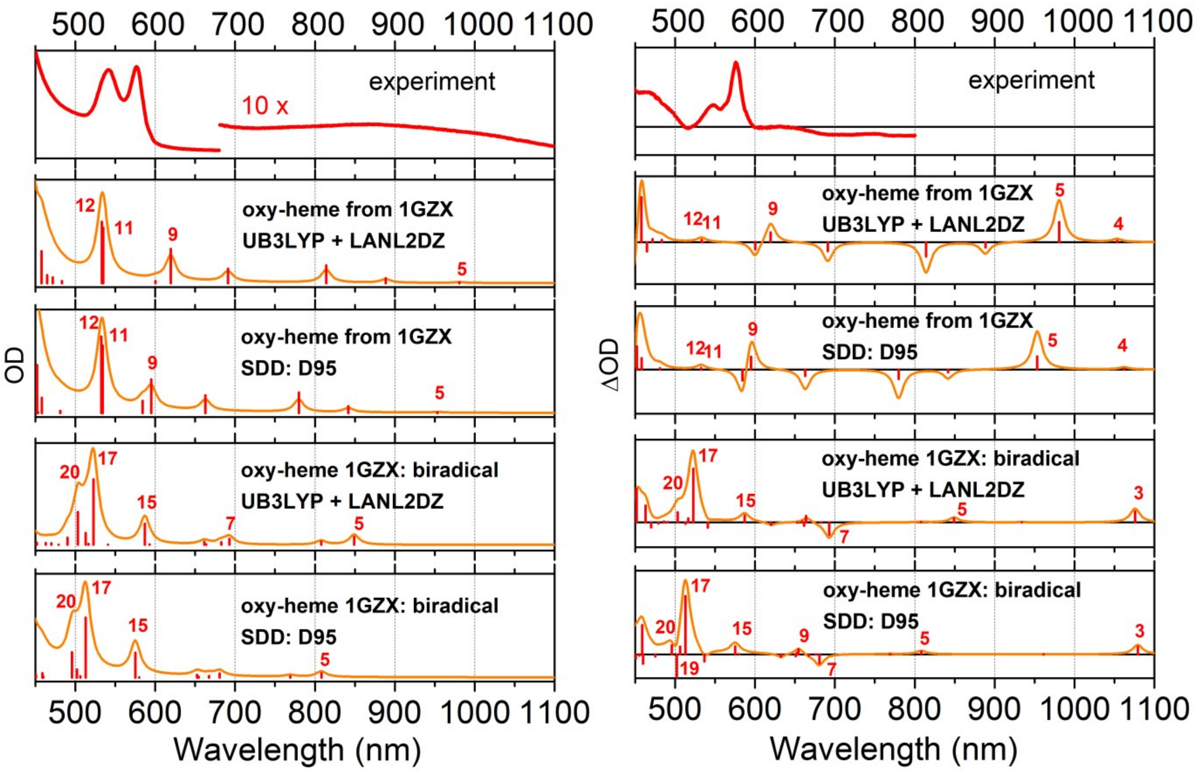

Fig. S3.

Optical spectroscopy in the visible spectral range and results of TD-DFT studies for oxy-Hb. Optical absorption (left) and circular dichroism (right) spectra; experimental and calculated for oxy-heme we extracted from 1GZX entry as reported in the Structural Bioinformatics Protein Data Bank using UB3LYP: 6-311++g(d,p)/LANL2DZ and SDD:D95 theory level. Numbers indicate NTO pairs.

To explore how possible structural variance may affect the electronic properties of oxy-Hb, in Fig. S4 we compare experimental optical absorption (left) and circular dichroism (right) spectra of oxy-Hb (i) where the oxygen molecule points toward histidine to form a hydrogen bond (Pauling geometry); (ii) the Pauling geometry biradical case; (iii) the Pauling geometry where

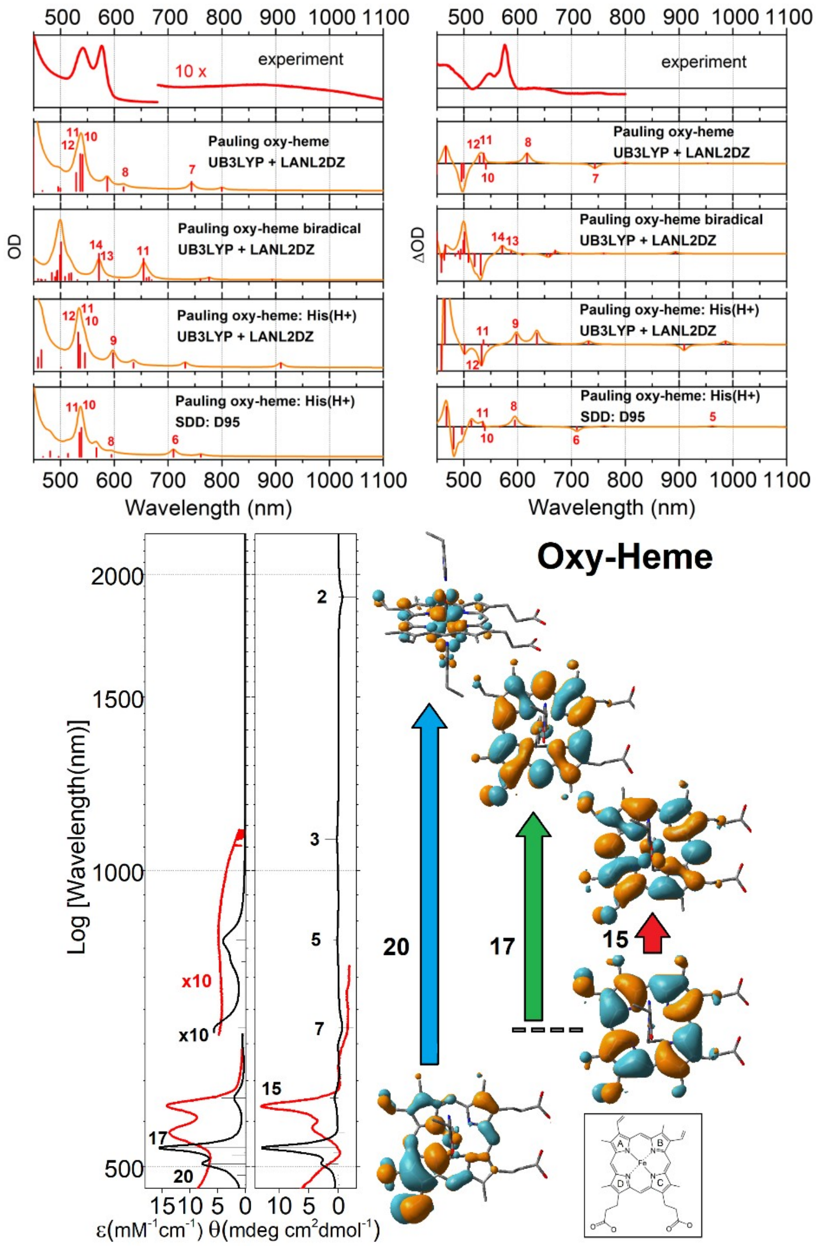

Fig. S4. Optical spectroscopy in the visible spectral range and results of TD-DFT studies for oxy-Hb. Upper panels: Optical absorption (left) and circular dichroism (right) spectra experimental and calculated for oxy-heme under the Pauling geometry, biradical under the Pauling geometry, the Pauling geometry case where the histidine next to the oxygen molecule is double protonated using UB3LYP: 6-311++g(d,p)/LANL2DZ. At the bottom we present optical spectra calculated under SDD:D95 theory using the structure of the Pauling geometry case where the histidine next to the oxygen molecule is double protonated as optimized under UB3LYP: 6-311++g(d,p)/LANL2DZ. Numbers indicate NTO pairs. **Lower panels:** Experimental (coloured lines) and theoretical (black lines) optical absorption and circular dichroism spectra for the biradical system at SDD:D95 theory; NTO pairs that account for the main spectral features in the visible spectral range. Here for simplicity, we sum contributions of α and β electrons.

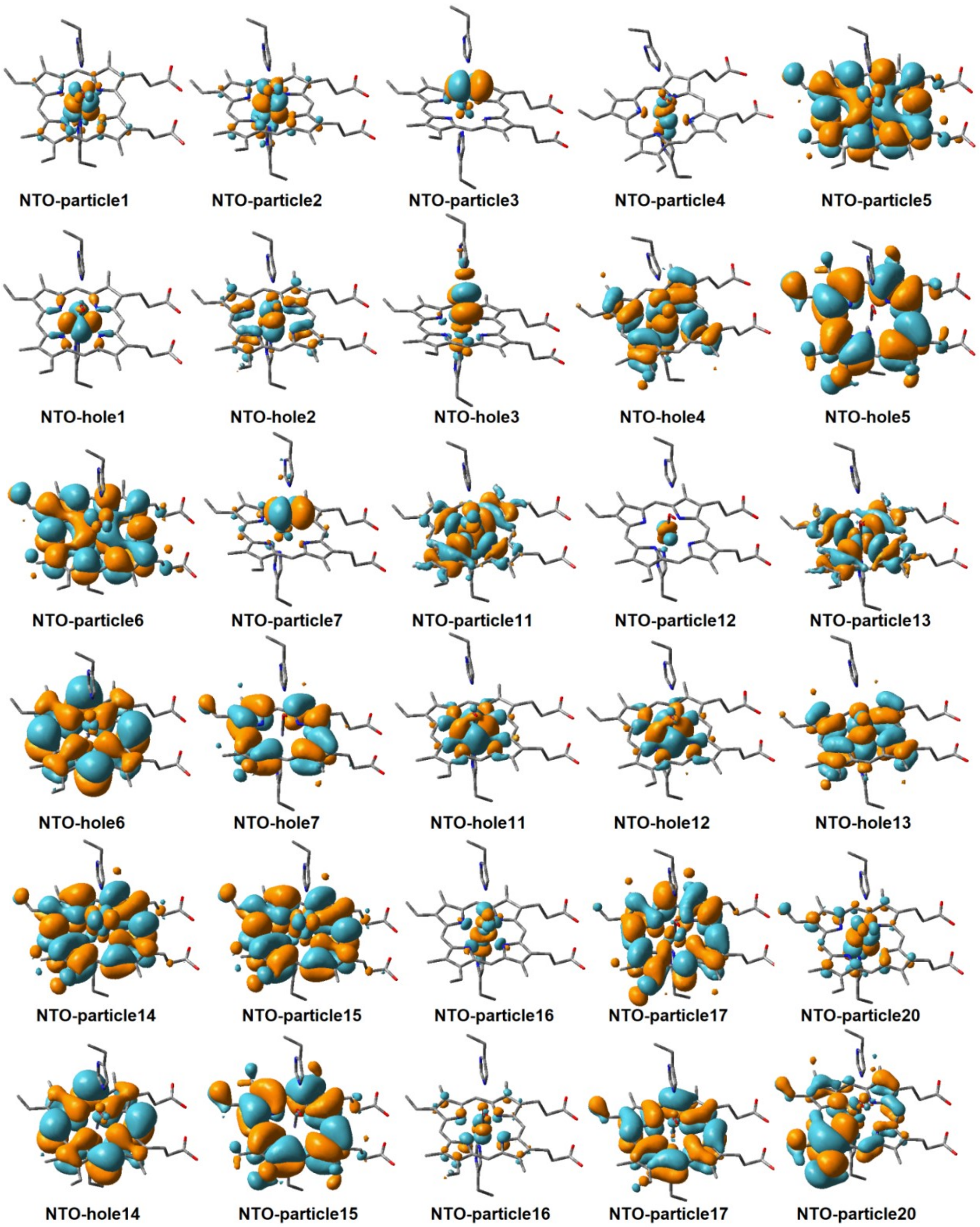

Fig. S5.

Electronic properties of oxy-heme. NTO pairs for the biradical oxy-heme structure extracted from 1GZX.pdb using UB3LYP: 6-311++g(d,p)/LANL2DZ. Pairs 1-4 contribute to optical absorption at 6805, 5381, 1312 and 747 nm. Pairs 11, 14 and 16 with resonances at 575, 551 and 489 nm determine optical absorption in the visible spectral range. In a case where α and β electrons participate in the optical transition, we sum their contributions properly scaled for simplicity in visual content. However, in the text, below, we quantify the contributions of α and β electrons.

the histidine next to the oxygen is double protonated. Here, finding a stable wavefunction for the biradical provides additional energy optimization of 14.8 kcal/mol. These three systems were optimized and computed using UB3LYP: 6-311++g(d,p)/LANL2DZ theory. Additionally, we conducted SDD:D95 theory computations of optical properties for the third structural case optimized under UB3LYP: 6-311++g(d,p)/LANL2DZ.

Comparing theoretical and experimental spectra in Fig. S3 and S4 we can clearly state that theoretical results for the biradical case (spectra in the lower panels in Fig. S3) computed either with UB3LYP: 6-311++g(d,p)/LANL2DZ or SDD:D95 theory show good agreement for the optical absorption and circular dichroism. Considering that (a) the structure is very close to that extracted from the X-ray reported 1GZX.pdb, (b) the energy of the bi-radical according to the stability of the wavefunction test, has the lowest value, (c) both theory levels show good agreement with the experimental, we can conclude that the NTO pairs 17 and 19 are the ones to describe the colour of oxy-Hb in blood in the visible spectral range. UB3LYP: 6-311++g(d,p)/LANL2DZ theory predicts the pairs to provide optical absorption at 522.6 and 513.1 nm, respectively: see Fig. S3. It is important to stress that d-d transitions of the iron do not play any significant role in both transitions.

In Fig. S5 we present selected NTO pairs for this structural case. We can clearly see that resonance 17 is dominated by the ligand electrons with a small contribution of ligand-to-metal charge transfer. At the same time, ligand-to-metal charge transfer dominates in pair 20 which is computed to provide absorption at 503.2 nm: see Fig. S3.

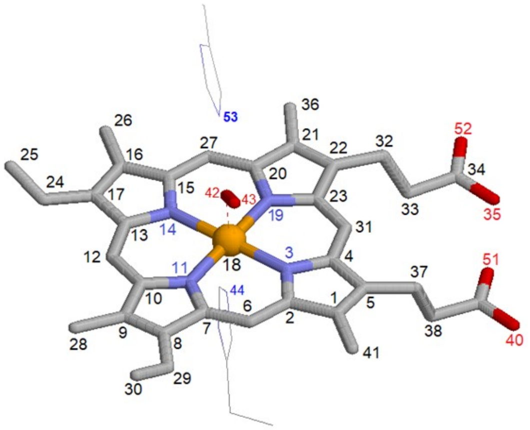

NTO pair 1: main localized contributions
Beta occ 216 OE=2.192 is Fe18-d=0.97
Beta vir 217 OE=2.192 is Fe18-d=0.89

NTO pair 2: main localized contributions
Beta occ 216 OE=2.047 is Fe18-d=0.88
Beta vir 217 OE=2.047 is Fe18-d=0.88

NTO pair 3: main localized contributions
Alpha occ 216 OE=1.996 is O43-p=0.51 O42-p=0.25 Fe18-d=0.07
Alpha vir 217 OE=1.996 is O43-p=0.54 O42-p=0.42

NTO pair 4: main localized contributions
Alpha occ 216 OE=0.797 is Fe18-d=0.88
Alpha vir 217 OE=0.797 is Fe18-d=0.65 O42-p=0.11 N44-p=0.05
Beta occ 216 OE=1.371 is Fe18-d=0.91
Beta vir 217 OE=1.371 is Fe18-d=0.74 O42-p=0.07 N44-p=0.05

NTO pair 15: main localized contributions
Alpha occ 216 OE=1.192 is C13-p=0.09 C7-p=0.09 C20-p=0.09 C10-p=0.09 C15-p=0.09 C4-p=0.09 C2-p=0.08 C23-p=0.08

Alpha vir 217 OE=1.192 is C12-p=0.14 C31-p=0.12 C15-p=0.06 C7-p=0.06 C2-p=0.06 C20-p=0.06 C9-p=0.05 C17-p=0.04
Beta occ 215 OE=0.319 is C31-p=0.15 C12-p=0.15 C6-p=0.14 C27-p=0.14 N19-p=0.07 N3-p=0.07 N14-p=0.07 N11-p=0.07
Beta occ 216 OE=0.464 is C10-p=0.09 C15-p=0.09 C23-p=0.09 C13-p=0.09 C2-p=0.09 C20-p=0.08 C4-p=0.08 C7-p=0.08
Beta vir 217 OE=0.464 is Fe18-d=0.67 C12-p=0.04
Beta vir 218 OE=0.319 is C27-p=0.12 C6-p=0.12 C4-p=0.08 C23-p=0.08 C13-p=0.07 C10-p=0.07 C1-p=0.05 C16-p=0.05 C21-p=0.04

NTO pair 17: main localized contributions
Alpha occ 216 OE=0.616 is C15-p=0.09 C2-p=0.09 C7-p=0.09 C20-p=0.08 C13-p=0.08 C10-p=0.08 C4-p=0.08 C23-p=0.08 C9-p=0.06 C17-p=0.06
Alpha vir 217 OE=0.616 is C27-p=0.13 C6-p=0.12 C23-p=0.08 C4-p=0.07 C10-p=0.06 C13-p=0.06 C21-p=0.05 C16-p=0.05 C8-p=0.04 C1-p=0.04
Beta occ 214 OE=0.318 is C9-p=0.18 N11-p=0.14 C16-p=0.10 N14-p=0.07 C30-p=0.05 Fe18-p=0.05 C8-p=0.04
Beta occ 215 OE=0.430 is C7-p=0.07 C13-p=0.07 C20-p=0.07 N11-p=0.07 C4-p=0.06 C8-p=0.06 C15-p=0.05 C10-p=0.05 C30-p=0.05 C2-p=0.05
Beta occ 216 OE=0.512 is C27-p=0.12 C12-p=0.11 C31-p=0.10 C6-p=0.10 C8-p=0.07 N19-p=0.06 N3-p=0.05 C30-p=0.05
Beta vir 217 OE=0.512 is C12-p=0.13 C31-p=0.09 Fe18-d=0.09 C9-p=0.07 C20-p=0.05 C15-p=0.05 C7-p=0.05 N14-p=0.05
Beta vir 218 OE=0.430 is Fe18-d=0.18 C6-p=0.10 C27-p=0.09 C4-p=0.07 C13-p=0.06 C1-p=0.06 C16-p=0.05 C23-p=0.05 N11-p=0.04
Beta vir 219 OE=0.318 is Fe18-d=0.58

NTO pair 20: main localized contributions
Alpha occ 215 OE=0.162 is C16-p=0.25 C25-p=0.09 C17-p=0.08 N14-p=0.05 C7-p=0.04 N3-p=0.04
Alpha occ 216 OE=0.371 is C9-p=0.19 C8-p=0.19 C30-p=0.18 N11-p=0.17 C29-p=0.06
Alpha vir 217 OE=0.371 is C9-p=0.15 C8-p=0.10 C30-p=0.09 C12-p=0.09 C6-p=0.07
Alpha vir 218 OE=0.162 is C16-p=0.12 C27-p=0.11 C17-p=0.08 C25-p=0.07 C6-p=0.05 C23-p=0.04 C4-p=0.04 C12-p=0.04
Beta occ 214 OE=0.242 is C31-p=0.14 C27-p=0.13 C12-p=0.12 C6-p=0.12 N14-p=0.12 N19-p=0.09 N3-p=0.08
Beta occ 215 OE=0.286 is C17-p=0.15 C25-p=0.13 N14-p=0.08 C16-p=0.07 Fe18-d=0.07 C9-p=0.05
Beta occ 216 OE=0.877 is C9-p=0.21 N11-p=0.17 C30-p=0.12 C8-p=0.11 C16-p=0.04 C29-p=0.04
Beta vir 217 OE=0.877 is Fe18-d=0.28 C9-p=0.10 C12-p=0.08 C7-p=0.05 C15-p=0.05 C20-p=0.05
Beta vir 218 OE=0.286 is Fe18-d=0.19 C27-p=0.10 C6-p=0.08 C23-p=0.07 C21-p=0.05 C16-p=0.05 C8-p=0.05
Beta vir 219 OE=0.242 is Fe18-d=0.34 C31-p=0.08 C12-p=0.06 C1-p=0.05 N11-p=0.04 N19-p=0.04

ELECTRONIC PROPERTIES OF MET-HB

Here, we compare experimental optical absorption (left) and circular dichroism (right) spectra of met-Hb with calculated spectra for deoxy-heme we prepared adding ligand water next to iron in

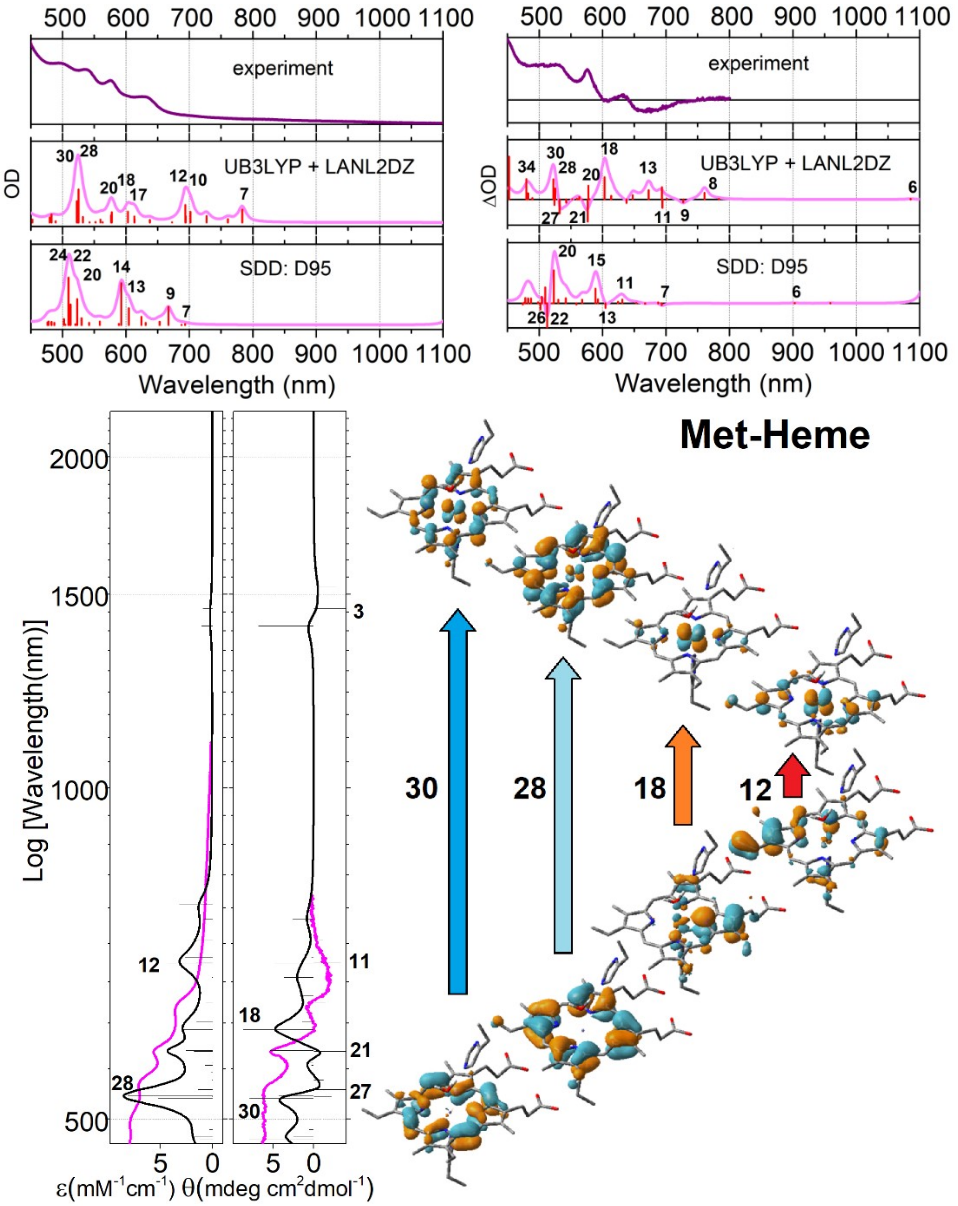

Fig. S6. Optical spectroscopy in the visible spectral range and results of TD-DFT studies for met-Hb. Upper panels: Optical absorption (left) and circular dichroism (right) spectra, experimental and calculated for met-heme using UB3LYP: 6-311++g(d,p)/LANL2DZ and SDD:D95 theory levels. Numbers indicate NTO pairs. **Lower panels:** Experimental (coloured lines) and theoretical (black lines) optical absorption and circular dichroism spectra for the system at SDD:D95 theory; NTO pairs that account for the main spectral features in the visible spectral range. Here for simplicity, we sum contributions of α and β electrons.

the structure extracted from 2HHB.pdb. Using UB3LYP: 6-311++g(d,p)/LANL2DZ we optimize structure and calculate optical absorption and circular dichroism spectra. Then, we repeat calculations of optical properties but using SDD:D95 theory for the structures optimized with UB3LYP: 6-311++g(d,p)/LANL2DZ.

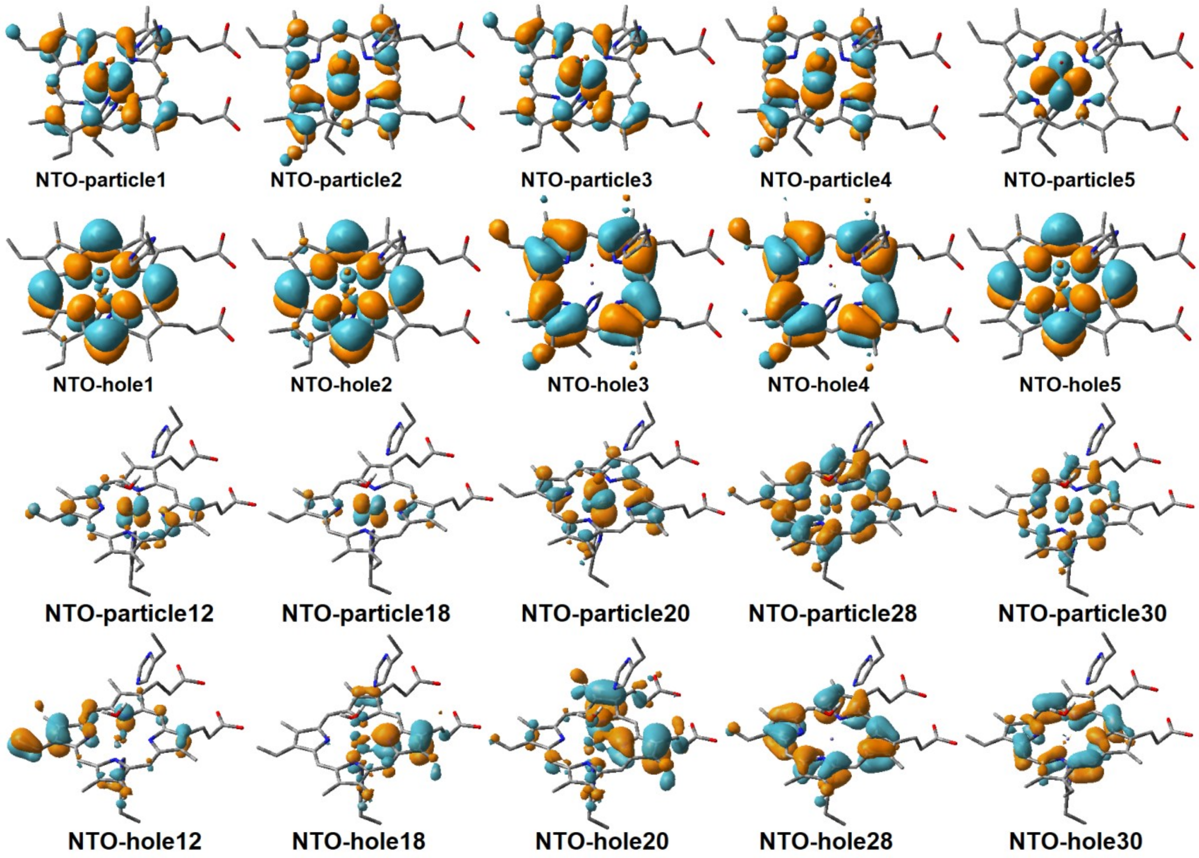

Fig. S7.

Electronic properties of met-heme. NTO pairs for met-heme using UB3LYP: 6-311++g(d,p)/LANL2DZ theory. Pairs 1-5 are to contribute optical absorption at 1582, 1522, 1456, 1404 and 1193 nm. Pairs 12, 18, 20, 28 and 30 with resonances at 693, 603, 577, 525 and 522 nm to determine optical absorption of the met-Hb in the visible spectral range. In a case if α and β electrons participate in the optical transition, we sum their contributions properly scaled for simplicity in visual content. However, in the text, below, we quantify the contributions of α and β electrons.

The calculated optical properties using UB3LYP: 6-311++g(d,p)/LANL2DZ resemble the experimental dispersions, save the main resonances in the visible spectral range demonstrate a relatively broader spread. We may suggest the calculated resonance of the 7th, 12th, 20th, 28th and 30th NTO pairs to represent the experimentally observed resonances at 650, 575, 540 and 500 nm, respectively. Such assignment is tentative because the level of theory used may not be high enough to describe well the nature of the numerous and dense electronic states that theory predicts for met-Hb in the visible spectral range. To confirm this we recalculated optical dispersions applying SDD:D95 theory level for the structure optimized using UB3LYP: 6-311++g(d,p)/LANL2DZ.

Indeed, under the higher level of theory, the spread of electronic resonances in the visible spectral range became comparable to that in experiment. To describe the nature of electronic transitions, in Fig. S7 we present images of the NTO pairs. We see that optical absorption of met-Hb in the visible spectral range is due to the ligand-to-metal charge transfer.

NTO pair 1: main localized contributions
Beta occ 210 OE=1.943 is C26-p=0.15 C8-p=0.14 C14-p=0.14 C20-p=0.13
Beta vir 211 OE=1.943 is Fe32-d=0.76
NTO pair 2: main localized contributions
Beta occ 209 OE=0.733 is C18-p=0.09
Beta occ 210 OE=1.257 is C8-p=0.14 C26-p=0.14 C14-p=0.14 C20-p=0.14
Beta vir 211 OE=1.257 is Fe32-d=0.74
Beta vir 212 OE=0.733 is Fe32-d=0.73

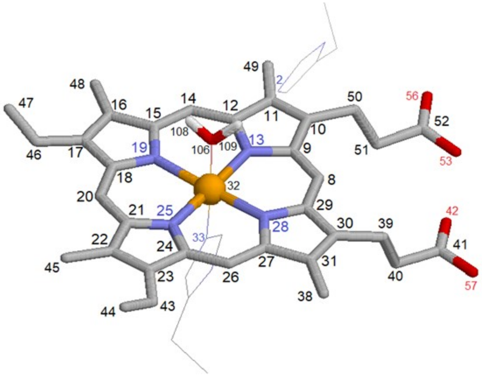

NTO pair 3: main localized contributions
Beta occ 209 OE=0.745 is C26-p=0.14 C8-p=0.14 C14-p=0.14 C20-p=0.13
Beta occ 210 OE=1.238 is C21-p=0.09
Beta vir 211 OE=1.238 is Fe32-d=0.75
Beta vir 212 OE=0.745 is Fe32-d=0.81

NTO pair 4: main localized contributions
Beta occ 210 OE=1.909 is C24-p=0.09
Beta vir 211 OE=1.909 is Fe32-d=0.75

NTO pair 5: main localized contributions
Beta occ 210 OE=1.999 is C26-p=0.14 C8-p=0.14 C14-p=0.14 C20-p=0.13
Beta vir 211 OE=1.999 is Fe32-d=0.97

NTO pair 6: main localized contributions
Beta occ 210 OE=1.999 is C18-p=0.09
Beta vir 211 OE=1.999 is Fe32-d=0.97

NTO pair 7: main localized contributions
Beta occ 210 OE=1.873 is N25-p=0.14 C22-p=0.13 N19-p=0.11 C23-p=0.11 C44-p=0.10
Beta vir 211 OE=1.873 is Fe32-d=0.76

NTO pair 8: main localized contributions
Beta occ 209 OE=0.697 is C22-p=0.12 N25-p=0.11 C16-p=0.11 N19-p=0.10
Beta occ 210 OE=1.255 is N19-p=0.13 N25-p=0.12 C22-p=0.11 C16-p=0.11
Beta vir 211 OE=1.255 is Fe32-d=0.76
Beta vir 212 OE=0.697 is Fe32-d=0.76

NTO pair 9: main localized contributions
Alpha occ 215 OE=0.100 is C20-p=0.13 C26-p=0.13 C14-p=0.12
Alpha vir 216 OE=0.100 is C14-p=0.13 C26-p=0.10

Beta occ 210 OE=1.736 is C16-p=0.14 N19-p=0.13 C47-p=0.11 C17-p=0.10
Beta vir 211 OE=1.736 is Fe32-d=0.77

NTO pair 10: main localized contributions
Alpha occ 214 OE=0.172 is C29-p=0.10
Alpha occ 215 OE=0.798 is C20-p=0.16 C8-p=0.16 C14-p=0.14 C26-p=0.14
Alpha vir 216 OE=0.798 is C20-p=0.13 C8-p=0.11
Alpha vir 217 OE=0.172 is C26-p=0.12 C14-p=0.11
Beta occ 208 OE=0.186 is C17-p=0.13 C47-p=0.12
Beta occ 209 OE=0.320 is C16-p=0.19
Beta occ 210 OE=0.457 is C20-p=0.16 C8-p=0.15 C14-p=0.12 C26-p=0.12 N19-p=0.11
Beta vir 211 OE=0.457 is Fe32-d=0.15 C20-p=0.12 C8-p=0.11
Beta vir 212 OE=0.320 is Fe32-d=0.68
Beta vir 213 OE=0.186 is Fe32-d=0.48

NTO pair 11: main localized contributions
Alpha occ 214 OE=0.400 is C21-p=0.08
Alpha occ 215 OE=0.548 is C26-p=0.12 C14-p=0.11
Alpha vir 216 OE=0.548 is C26-p=0.14 C14-p=0.14
Alpha vir 217 OE=0.400 is C20-p=0.14 C8-p=0.12
Beta occ 207 OE=0.101 is C16-p=0.16 N19-p=0.12
Beta occ 208 OE=0.210 is C24-p=0.09
Beta occ 209 OE=0.333 is C14-p=0.12 C26-p=0.12 N25-p=0.11
Beta occ 210 OE=0.417 is C22-p=0.15
Beta vir 211 OE=0.417 is Fe32-d=0.84
Beta vir 212 OE=0.333 is Fe32-d=0.15
Beta vir 213 OE=0.210 is Fe32-d=0.44
Beta vir 214 OE=0.101 is Fe32-d=0.27

NTO pair 12: main localized contributions
Alpha occ 215 OE=0.452 is C14-p=0.08
Alpha vir 216 OE=0.452 is C14-p=0.11 C26-p=0.11
Beta occ 208 OE=0.137 is C26-p=0.17 C14-p=0.16 C20-p=0.11 C8-p=0.11
Beta occ 209 OE=0.452 is C22-p=0.10 N13-p=0.10
Beta occ 210 OE=0.779 is C22-p=0.14 C16-p=0.12
Beta vir 211 OE=0.779 is Fe32-d=0.78
Beta vir 212 OE=0.452 is Fe32-d=0.75
Beta vir 213 OE=0.137 is C14-p=0.14 C26-p=0.13 Fe32-d=0.12

NTO pair 17: main localized contributions
Alpha occ 214 OE=0.177 is C26-p=0.15 C14-p=0.15 C20-p=0.14 C8-p=0.14
Alpha occ 215 OE=0.542 is C15-p=0.09
Alpha vir 216 OE=0.542 is C20-p=0.09
Alpha vir 217 OE=0.177 is C26-p=0.08
Beta occ 208 OE=0.239 is C26-p=0.16 C8-p=0.15 C14-p=0.14 C20-p=0.13 N28-p=0.10
Beta occ 209 OE=0.437 is C18-p=0.09

Beta occ 210 OE=0.600 is N28-p=0.25 C30-p=0.24 C31-p=0.23
Beta vir 211 OE=0.600 is Fe32-d=0.74
Beta vir 212 OE=0.437 is Fe32-d=0.15
Beta vir 213 OE=0.239 is Fe32-d=0.13

NTO pair 18: main localized contributions
Alpha occ 214 OE=0.129 is C12-p=0.09
Alpha occ 215 OE=0.174 is C20-p=0.15 C26-p=0.14 C8-p=0.14 C14-p=0.14
Alpha vir 216 OE=0.174 is C20-p=0.14 C8-p=0.12
Alpha vir 217 OE=0.129 is C14-p=0.13 C26-p=0.13
Beta occ 207 OE=0.169 is C21-p=0.09
Beta occ 208 OE=0.199 is C20-p=0.13 C26-p=0.12 C8-p=0.12 C14-p=0.11
Beta occ 209 OE=0.519 is C30-p=0.13 C31-p=0.12 N28-p=0.11
Beta occ 210 OE=0.804 is N13-p=0.18 C11-p=0.17 C10-p=0.14 N28-p=0.11
Beta vir 211 OE=0.804 is Fe32-d=0.78
Beta vir 212 OE=0.519 is Fe32-d=0.76
Beta vir 213 OE=0.199 is Fe32-d=0.29
Beta vir 214 OE=0.169 is C26-p=0.11 C14-p=0.11

NTO pair 19: main localized contributions
Beta occ 210 OE=1.986 is C4-p=0.32 C3-p=0.29 C1-p=0.25
Beta vir 211 OE=1.986 is Fe32-d=0.75

NTO pair 20: main localized contributions
Beta occ 208 OE=0.114 is C26-p=0.15 C14-p=0.14 C8-p=0.14 C20-p=0.13
Beta occ 209 OE=0.202 is C21-p=0.09
Beta occ 210 OE=1.556 is C30-p=0.16 C31-p=0.13 C10-p=0.13 N28-p=0.12
Beta vir 211 OE=1.556 is Fe32-d=0.76
Beta vir 212 OE=0.202 is Fe32-d=0.12 C20-p=0.12 C8-p=0.12
Beta vir 213 OE=0.114 is Fe32-d=0.53

NTO pair 21: main localized contributions
Beta occ 209 OE=0.416 is C10-p=0.17 C11-p=0.14 N13-p=0.12 C30-p=0.12
Beta occ 210 OE=1.487 is C26-p=0.14 C8-p=0.14 C14-p=0.14 C20-p=0.13 N28-p=0.10
Beta vir 211 OE=1.487 is Fe32-d=0.75
Beta vir 212 OE=0.416 is Fe32-d=0.77

NTO pair 22: main localized contributions
Beta occ 210 OE=1.999 is C4-p=0.32 C3-p=0.29 C1-p=0.26
Beta vir 211 OE=1.999 is Fe32-d=0.75

NTO pair 23: main localized contributions
Beta occ 210 OE=1.938 is N28-p=0.17 N13-p=0.17 C11-p=0.14 C31-p=0.14 C30-p=0.11
Beta vir 211 OE=1.938 is Fe32-d=0.97

NTO pair 24: main localized contributions

Beta occ 208 OE=0.179 is C26-p=0.14 C8-p=0.13 C14-p=0.13 C20-p=0.13
Beta occ 209 OE=0.215 is C11-p=0.22 N13-p=0.22 C10-p=0.17
Beta occ 210 OE=1.546 is C35-p=0.29 C34-p=0.23 C37-p=0.23
Beta vir 211 OE=1.546 is Fe32-d=0.76
Beta vir 212 OE=0.215 is Fe32-d=0.82
Beta vir 213 OE=0.179 is Fe32-d=0.74

NTO pair 25: main localized contributions
Beta occ 210 OE=1.985 is C18-p=0.09
Beta vir 211 OE=1.985 is Fe32-d=0.75

NTO pair 26: main localized contributions
Beta occ 210 OE=1.854 is C35-p=0.30 C34-p=0.28 C37-p=0.23
Beta vir 211 OE=1.854 is Fe32-d=0.78

NTO pair 27: main localized contributions
Alpha occ 214 OE=0.109 is C14-p=0.15 C26-p=0.14 C8-p=0.14
Alpha occ 215 OE=0.125 is C27-p=0.09
Alpha vir 216 OE=0.125 is C26-p=0.13 C14-p=0.12
Alpha vir 217 OE=0.109 is C20-p=0.13 C8-p=0.10
Beta occ 207 OE=0.226 is C26-p=0.09
Beta occ 208 OE=0.260 is N13-p=0.14
Beta occ 209 OE=0.290 is C10-p=0.19
Beta occ 210 OE=0.961 is C34-p=0.23 C35-p=0.21 C37-p=0.14
Beta vir 211 OE=0.961 is Fe32-d=0.76
Beta vir 212 OE=0.290 is Fe32-d=0.45
Beta vir 213 OE=0.260 is Fe32-d=0.56
Beta vir 214 OE=0.226 is Fe32-d=0.31

NTO pair 28: main localized contributions
Alpha occ 214 OE=0.315 is C8-p=0.13 C20-p=0.13
Alpha occ 215 OE=0.349 is C21-p=0.08
Alpha vir 216 OE=0.349 is C20-p=0.12 C8-p=0.10
Alpha vir 217 OE=0.315 is C14-p=0.12 C26-p=0.11
Beta occ 209 OE=0.576 is C8-p=0.10
Beta occ 210 OE=0.624 is C21-p=0.08
Beta vir 211 OE=0.624 is C20-p=0.12
Beta vir 212 OE=0.576 is C14-p=0.11 C26-p=0.11

NTO pair 29: main localized contributions
Beta occ 210 OE=1.985 is C10-p=0.15 C30-p=0.13 C11-p=0.12 C31-p=0.11
Beta vir 211 OE=1.985 is Fe32-d=0.97

NTO pair 30: main localized contributions
Alpha occ 214 OE=0.222 is C24-p=0.10
Alpha occ 215 OE=0.263 is C14-p=0.16 C26-p=0.16 C20-p=0.13 C8-p=0.12

Alpha vir 216 OE=0.263 is C20-p=0.11
Alpha vir 217 OE=0.222 is C14-p=0.11 C26-p=0.11
Beta occ 207 OE=0.144 is C11-p=0.22 N13-p=0.21 C10-p=0.18
Beta occ 208 OE=0.380 is C21-p=0.09
Beta occ 209 OE=0.420 is N28-p=0.19 C30-p=0.19 C31-p=0.13
Beta occ 210 OE=0.555 is C14-p=0.15 C26-p=0.15 C20-p=0.14 C8-p=0.13
Beta vir 211 OE=0.555 is Fe32-d=0.20 C20-p=0.11
Beta vir 212 OE=0.420 is Fe32-d=0.71
Beta vir 213 OE=0.380 is C14-p=0.13 C26-p=0.12
Beta vir 214 OE=0.144 is Fe32-d=0.78

<u>COMPARISONS OF COMPUTED RAMAN SPECTRA WITH EXPERIMENTAL RESULTS IN THE BULK</u>

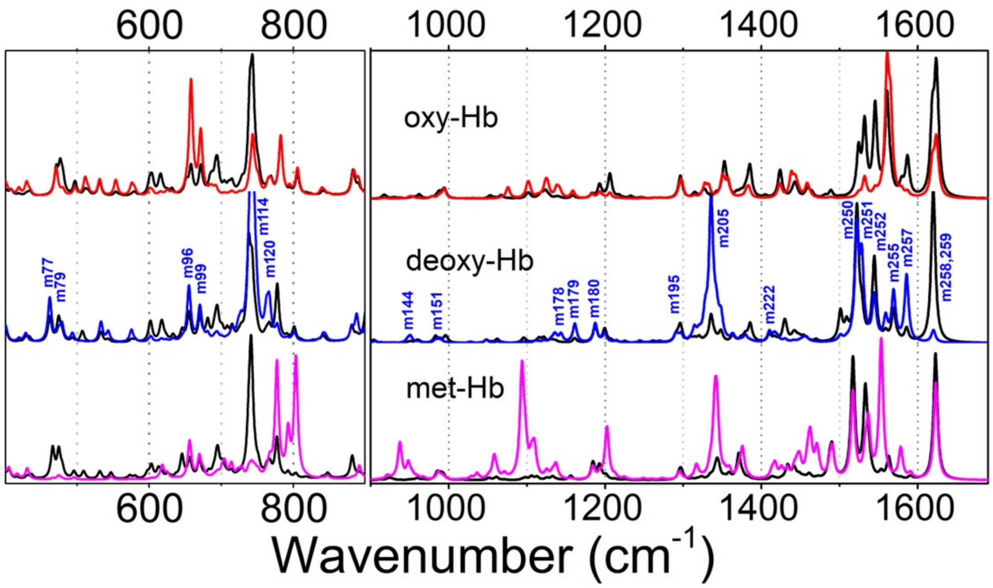

Fig. S8.

Theoretical Raman studies in the considered heme systems. Comparisons of DFT predictions for non-resonant (black lines) and resonant (coloured lines) Raman responses of oxy-heme (biradical), deoxy-heme and met heme, as indicated. The amplitude of resonant Raman spectra are scaled down by a factor of 1000. Frequencies of the computed resonances are scaled by 0.97.

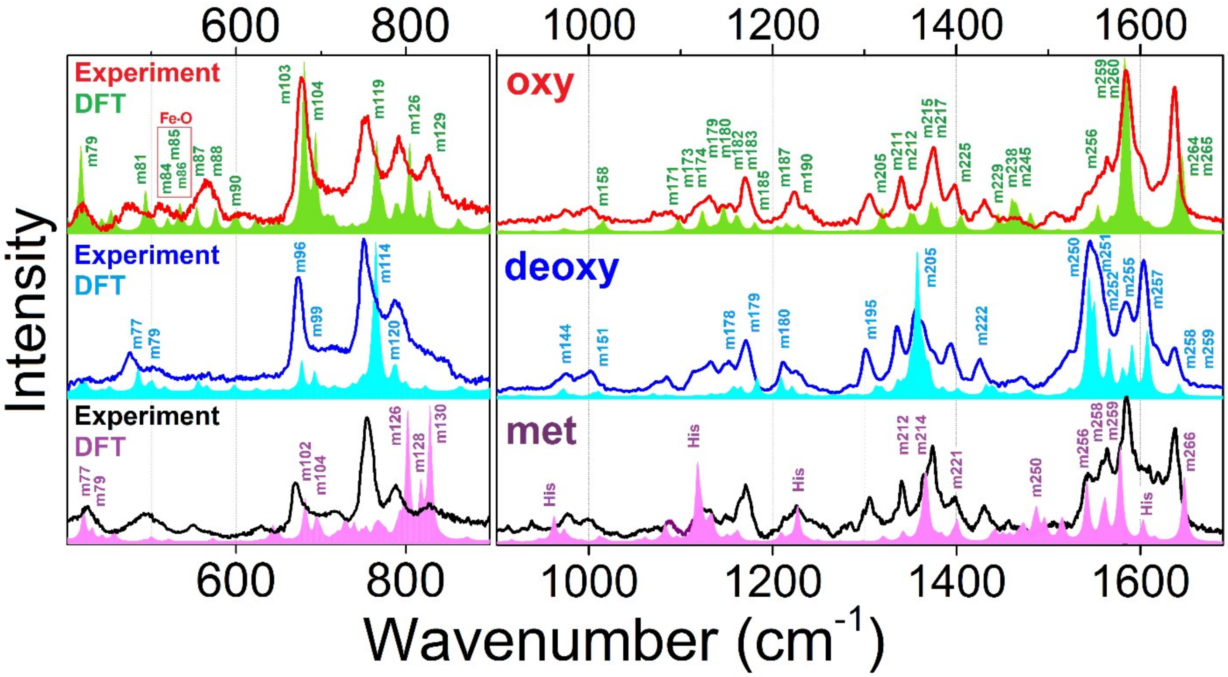

Fig. S9.

Comparisons of theory predicted Raman for the three forms of heme with experimental responses of the corresponding hemoglobins. The experimental spectra are measured in water droplets of prepared hemoglobins.

<u>**IMAGES OF ATOMIC DISPLACEMENTS OF MAIN NORMAL MODES COMPUTED FOR THE OXY-HEME BIRADICAL**</u>

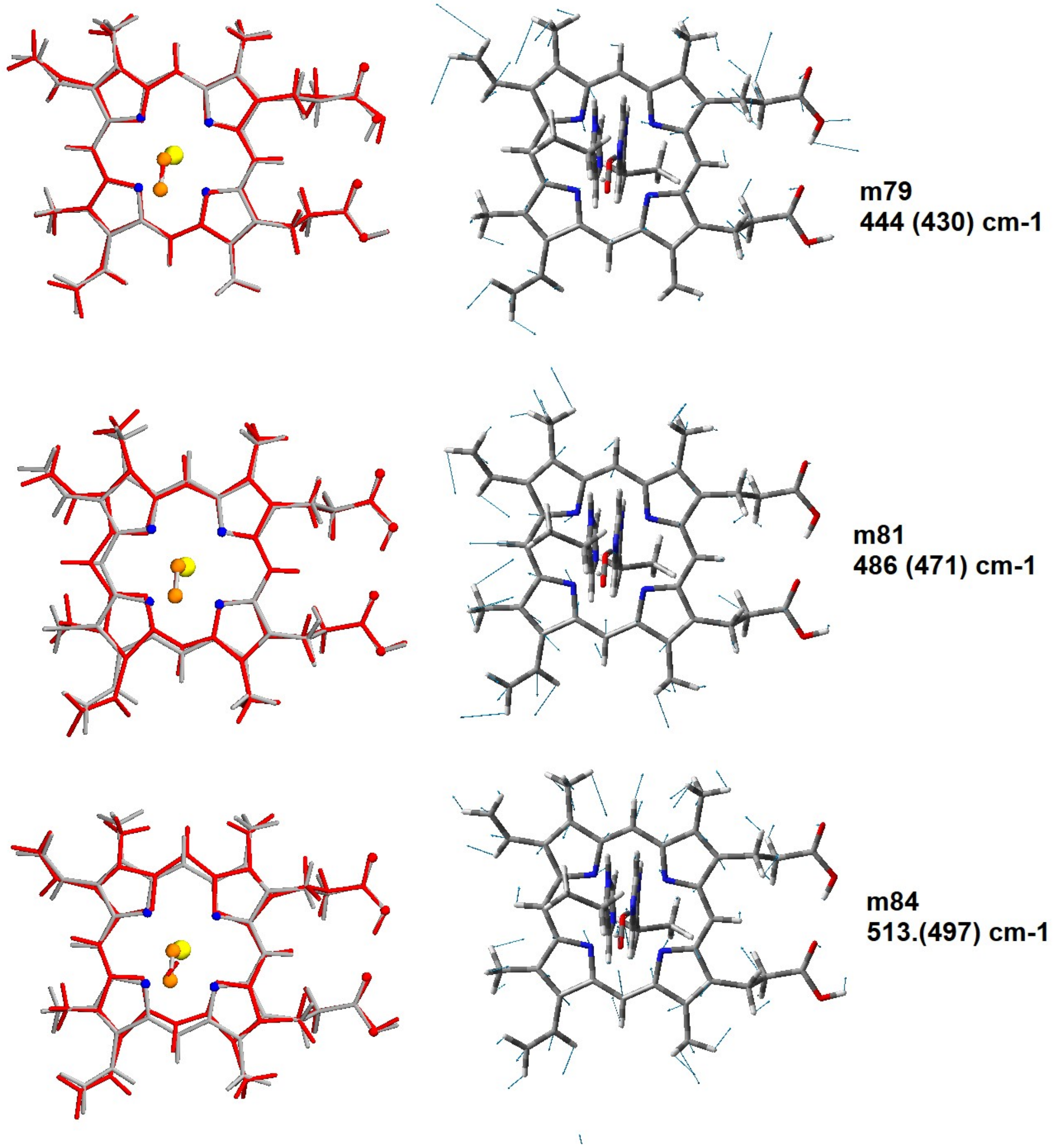

Fig. S10.

Graphical presentations of the normal modes (as indicated) that contribute to the resonant Raman of the oxy-Hb biradical. Left**:** structural changes (red lines) along the normal modes about the equilibrium structure (grey line): to help visualization, we remove histidines. Right**:** displacement vectors mapped on the equilibrium structure of the oxy-Hb biradical. Numbers at the right show the mode number and the frequency as printed by G09 package and scaled by 0.97 (in brackets) to assist visual inspection of resonances in Fig. S8 and S9.

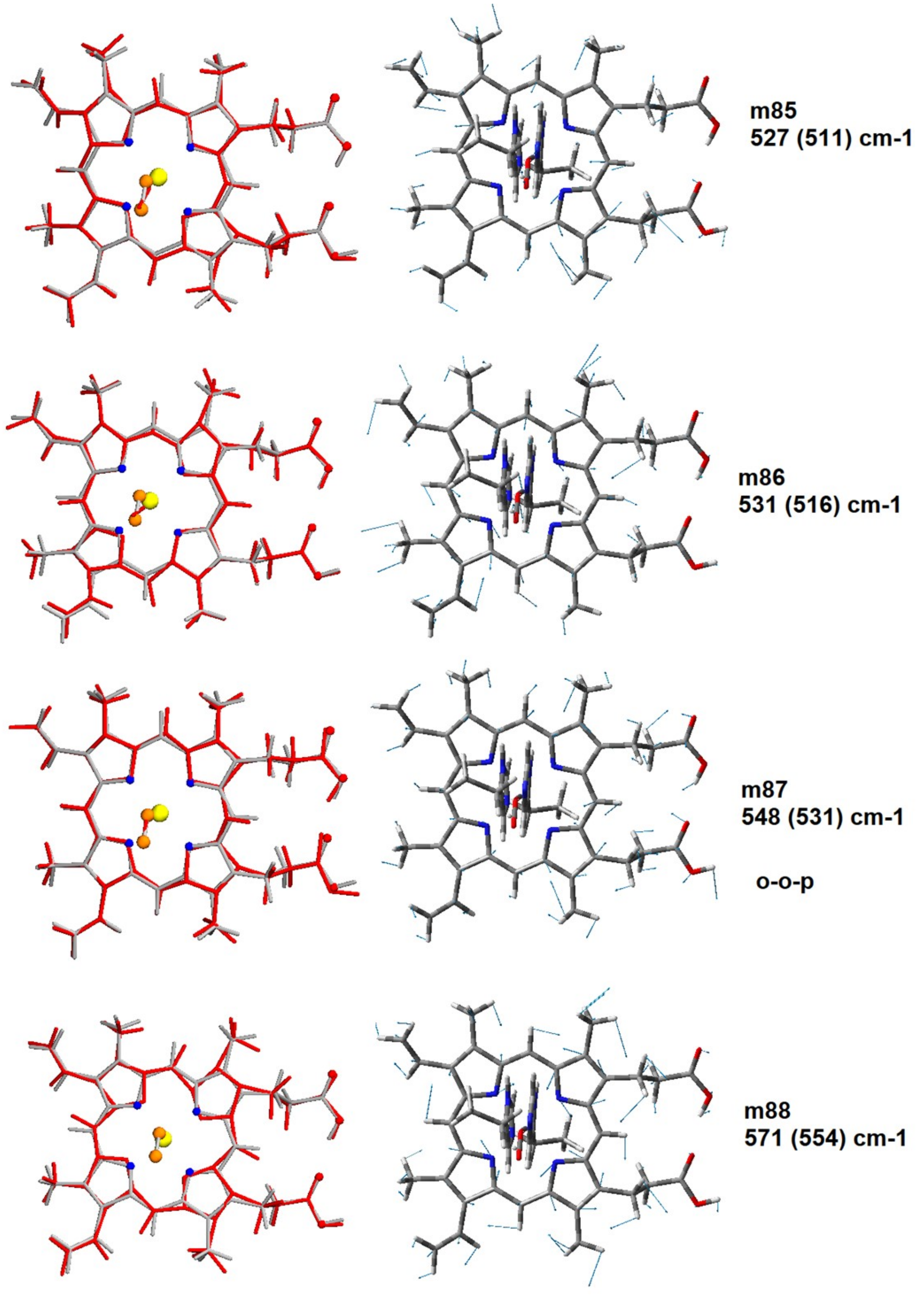

Fig. S11.

Graphical presentations of the normal modes (as indicated) that contribute to the resonant Raman of the oxy-Hb biradical. Left**:** structural changes (red lines) along the normal modes about the equilibrium structure (grey line): to help visualization, we remove histidines. Right**:** displacement vectors mapped on the equilibrium structure of the oxy-Hb biradical. Numbers at the right show the mode number and the frequency as printed by G09 package and scaled by 0.97 (in brackets) to assist visual inspection of resonances in Fig. S8 and S9.

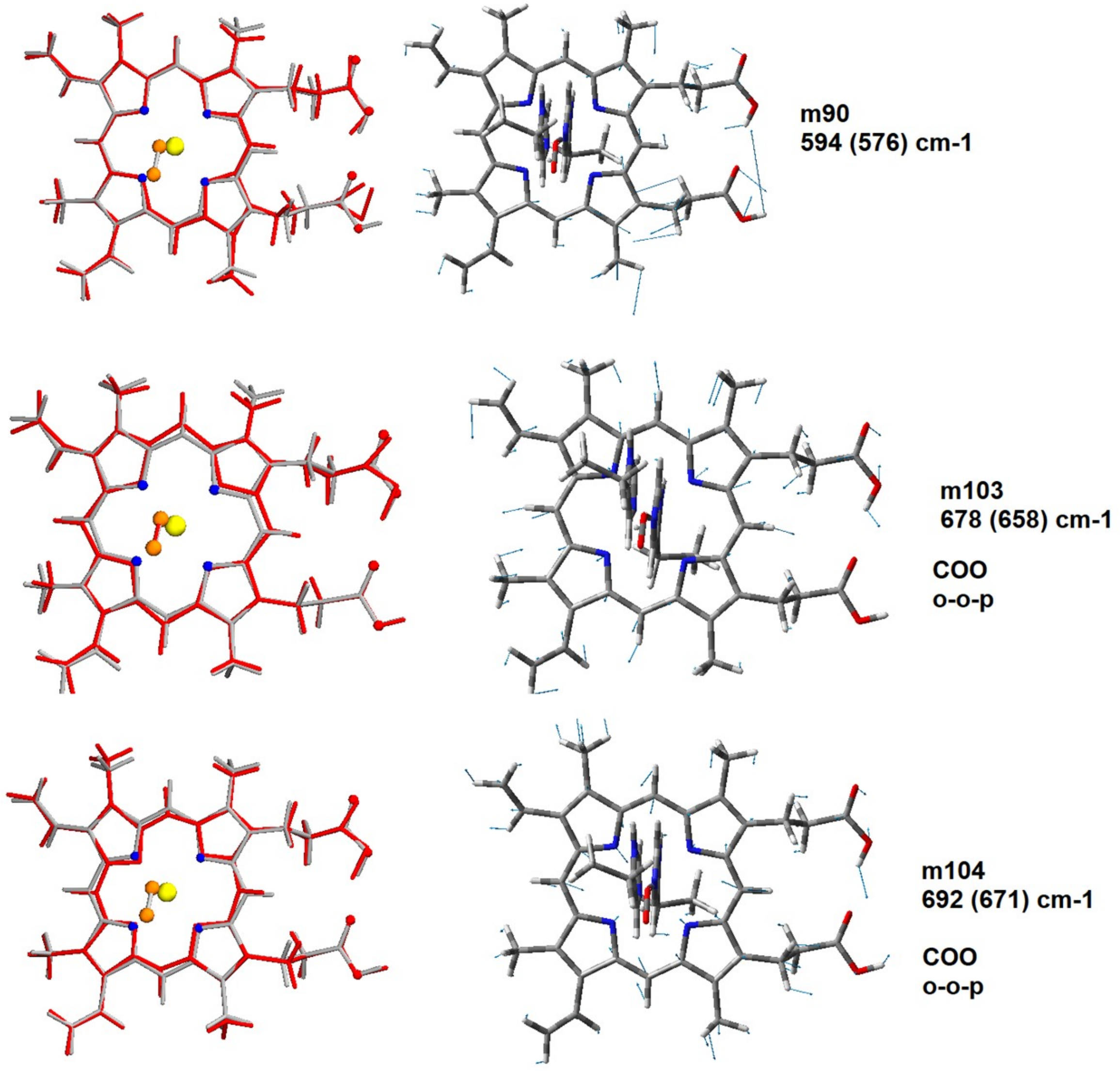

Fig. S12.

Graphical presentations of the normal modes (as indicated) that contribute to the resonant Raman of the oxy-Hb biradical. Left**:** structural changes (red lines) along the normal modes about the equilibrium structure (grey line): to help visualization, we remove histidines. Right**:** displacement vectors mapped on the equilibrium structure of the oxy-Hb biradical. Numbers at the right show the mode number and the frequency as printed by G09 package and scaled by 0.97 (in brackets) to assist visual inspection of resonances in Fig. S8 and S9.

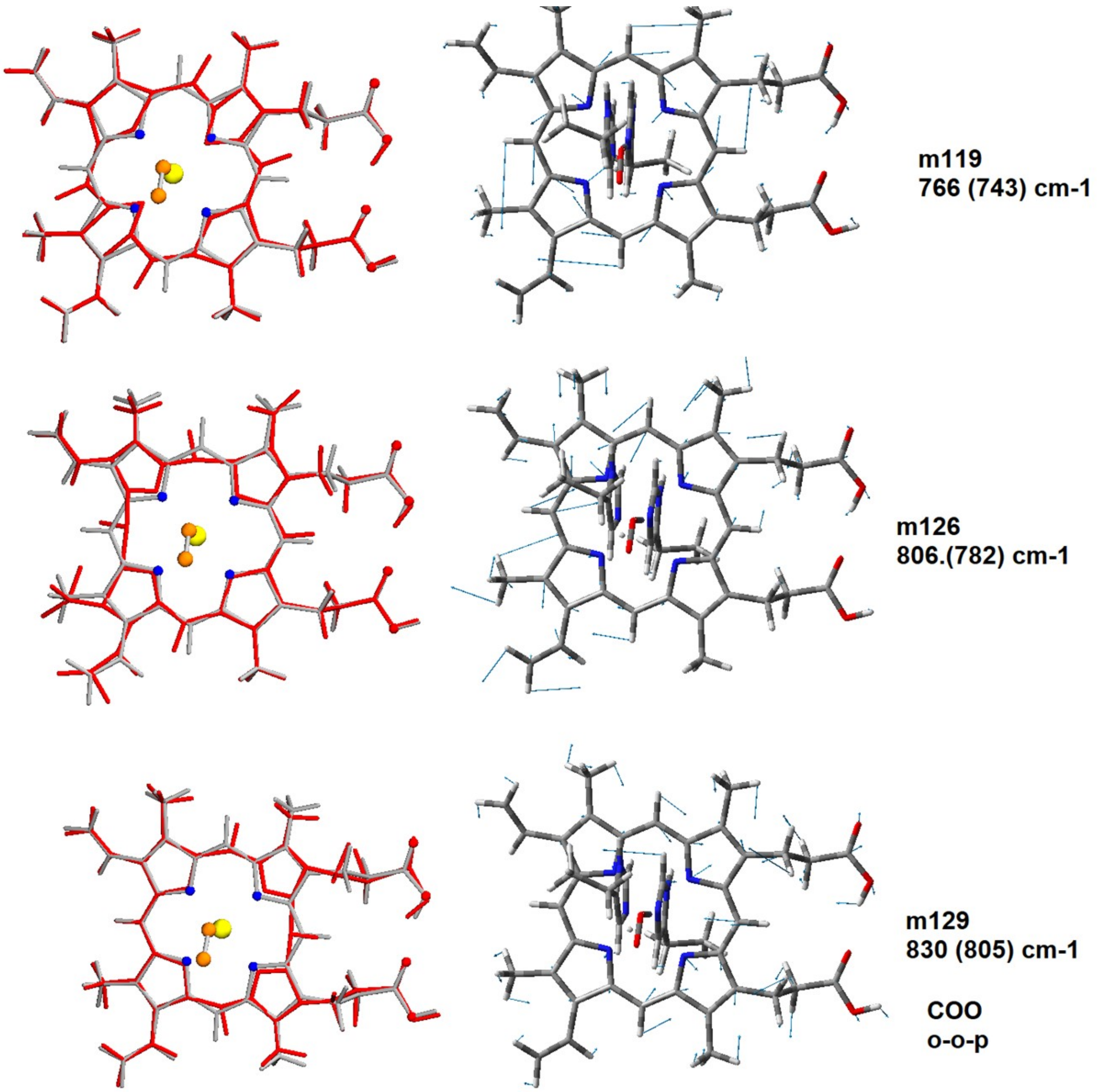

Fig. S13.

Graphical presentations of the normal modes (as indicated) that contribute to the resonant Raman of the oxy-Hb biradical. Left**:** structural changes (red lines) along the normal modes about the equilibrium structure (grey line): to help visualization, we remove histidines. Right**:** displacement vectors mapped on the equilibrium structure of the oxy-Hb biradical. Numbers at the right show the mode number and the frequency as printed by G09 package and scaled by 0.97 (in brackets) to assist visual inspection of resonances in Fig. S8 and S9.

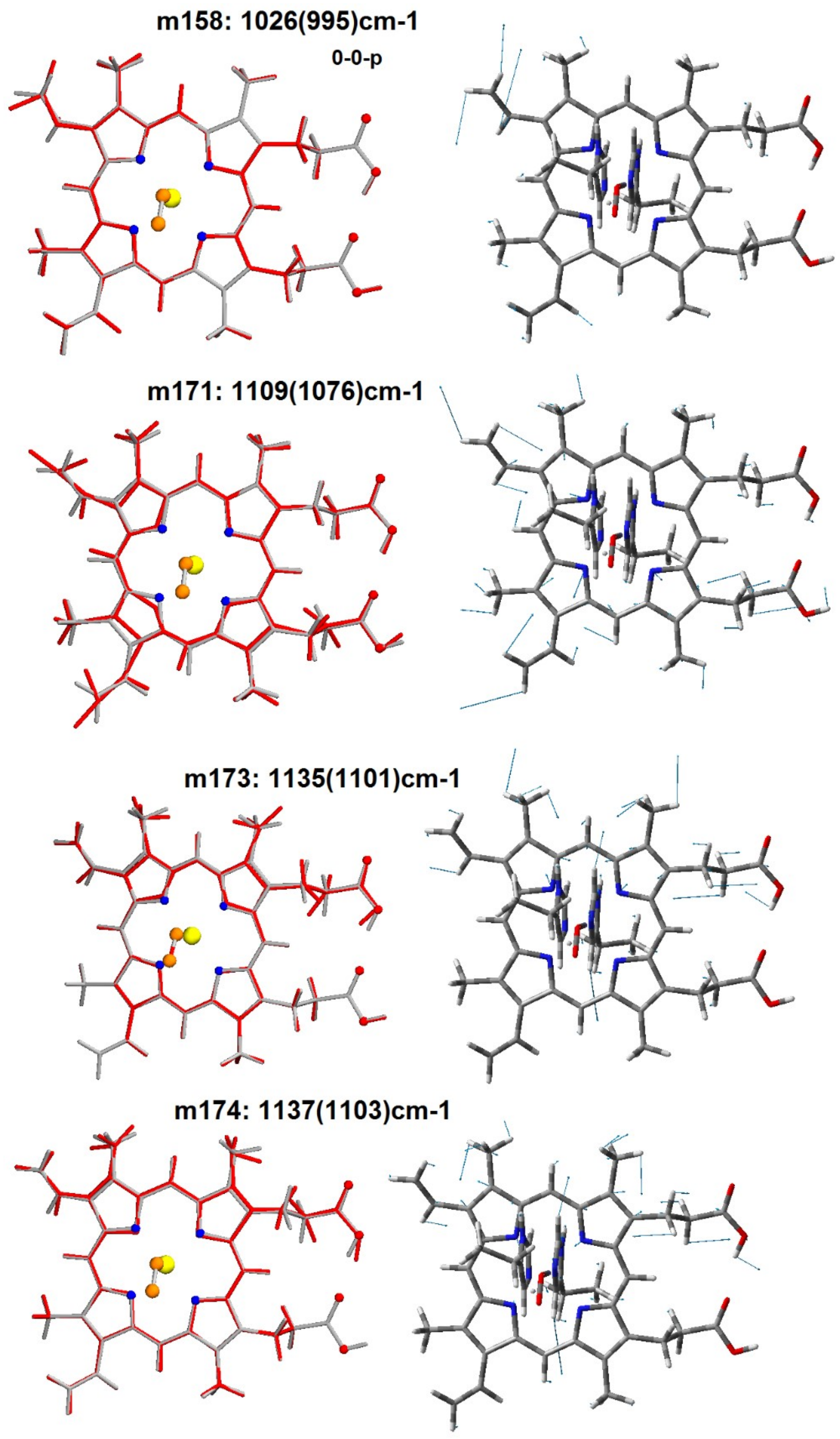

Fig. S14.

Graphical presentations of the normal modes (as indicated) that contribute to the resonant Raman of the oxy-Hb biradical. Left**:** structural changes (red lines) along the normal modes about the equilibrium structure (grey line): to help visualization, we remove histidines. Right**:** displacement vectors mapped on the equilibrium structure of the oxy-Hb biradical.

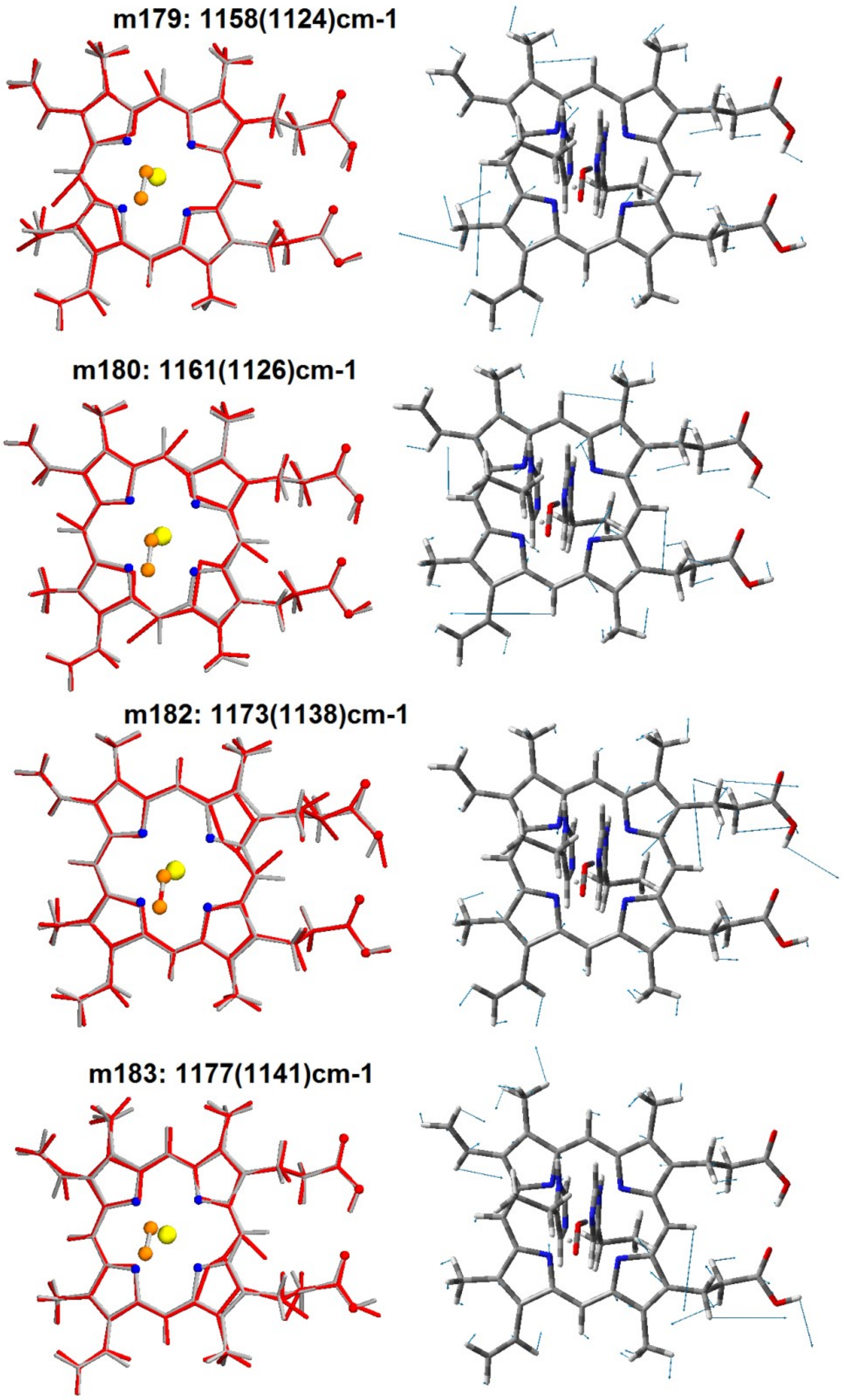

Fig. S15.

Graphical presentations of the normal modes (as indicated) that contribute to the resonant Raman of the oxy-Hb biradical. Left**:** structural changes (red lines) along the normal modes about the equilibrium structure (grey line): to help visualization, we remove histidines. Right**:** displacement vectors mapped on the equilibrium structure of the oxy-Hb biradical.

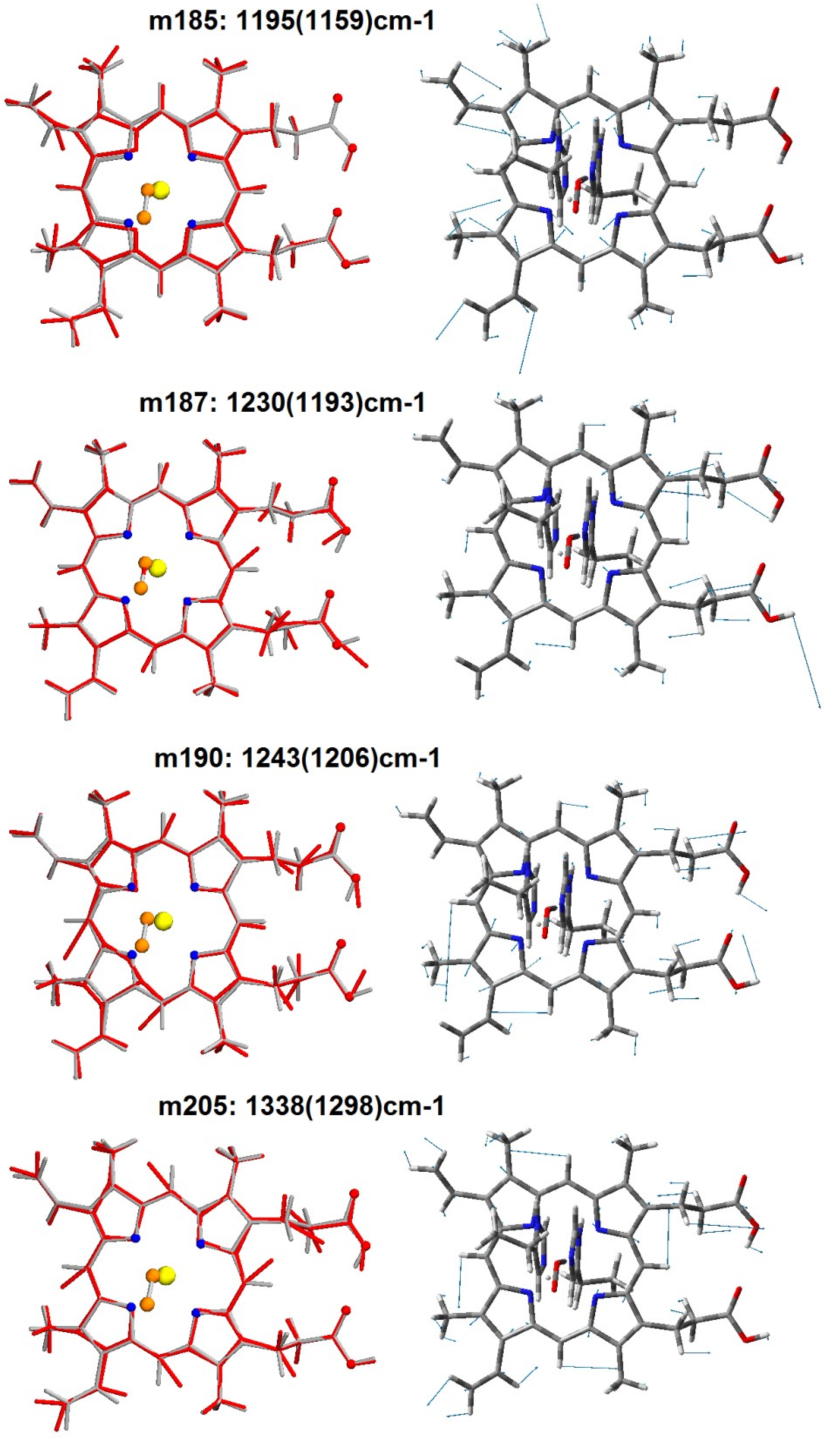

Fig. S16.

Graphical presentations of the normal modes (as indicated) that contribute to the resonant Raman of the oxy-Hb biradical. Left**:** structural changes (red lines) along the normal modes about the equilibrium structure (grey line): to help visualization, we remove histidines. Right**:** displacement vectors mapped on the equilibrium structure of the oxy-Hb biradical.

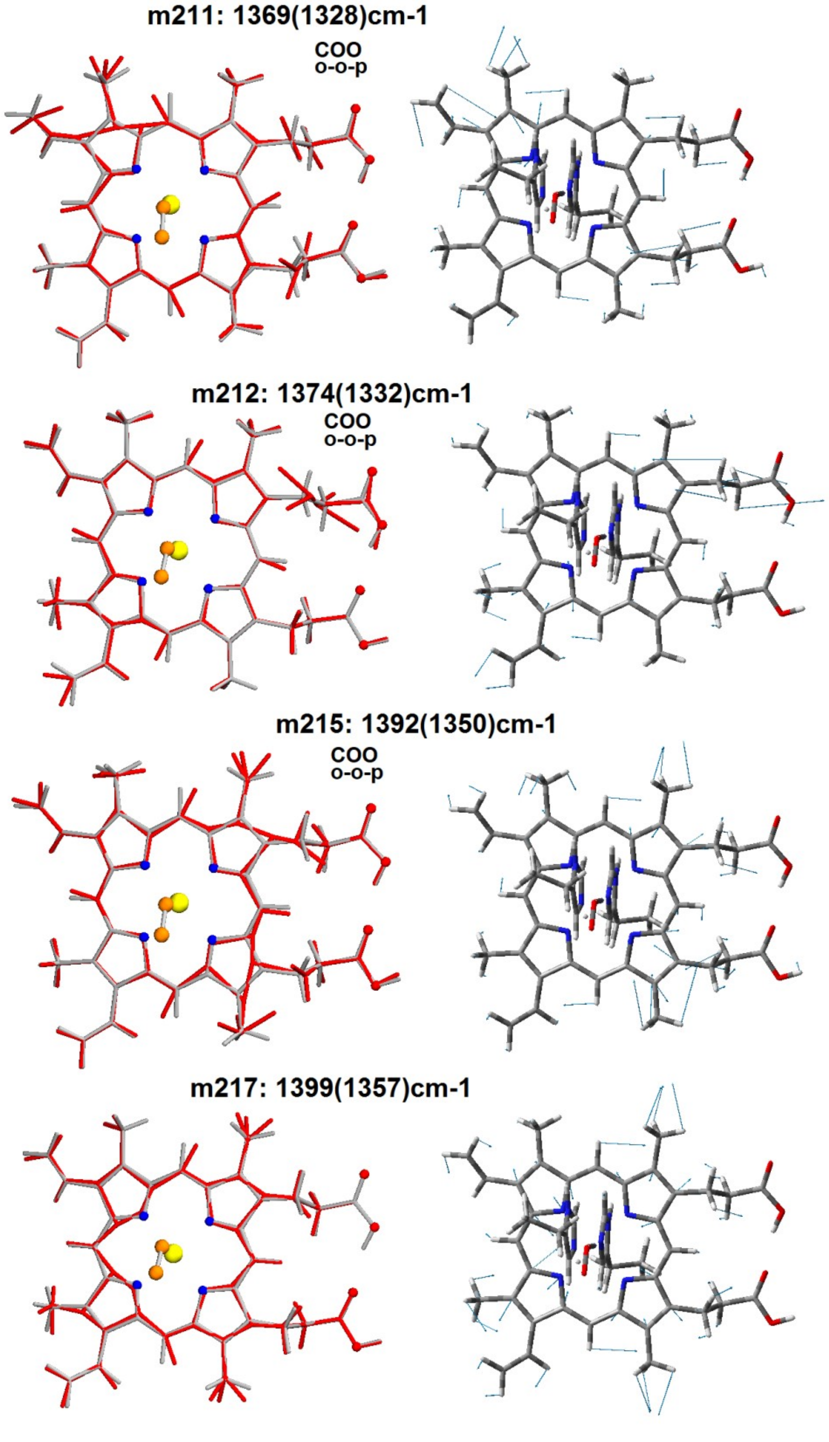

Fig. S17.

Graphical presentations of the normal modes (as indicated) that contribute to the resonant Raman of oxy-Hb. Left**:** structural changes (red lines) along the normal modes about the equilibrium structure (grey line): to help visualization, we remove histidines. Right**:** displacement vectors mapped on the equilibrium structure of oxy-Hb.

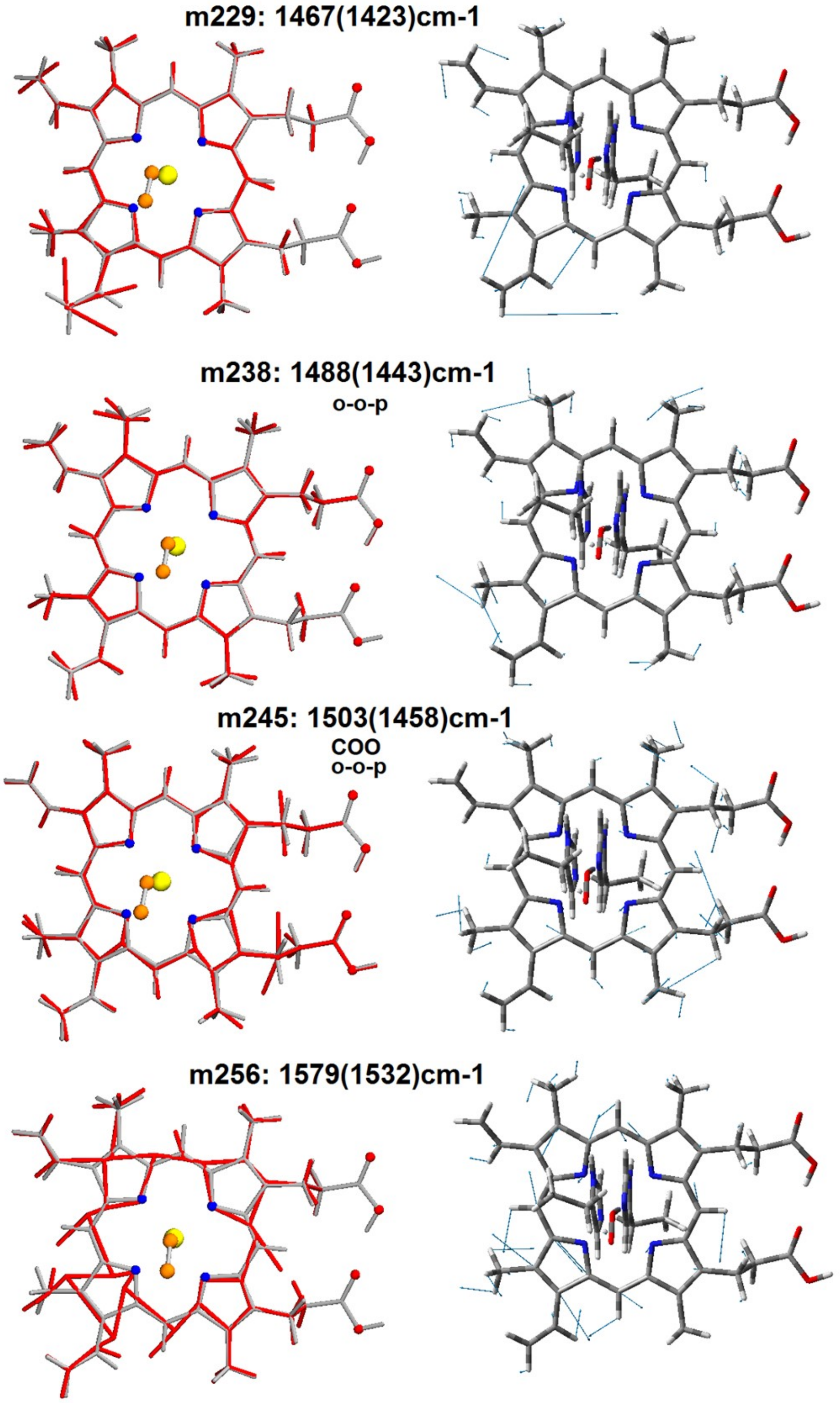

Fig. S18.

Graphical presentations of the normal modes (as indicated) that contribute to the resonant Raman of the oxy-Hb biradical. Left**:** structural changes (red lines) along the normal modes about the equilibrium structure (grey line): to help visualization, we remove histidines. Right**:** displacement vectors mapped on the equilibrium structure of the oxy-Hb biradical.

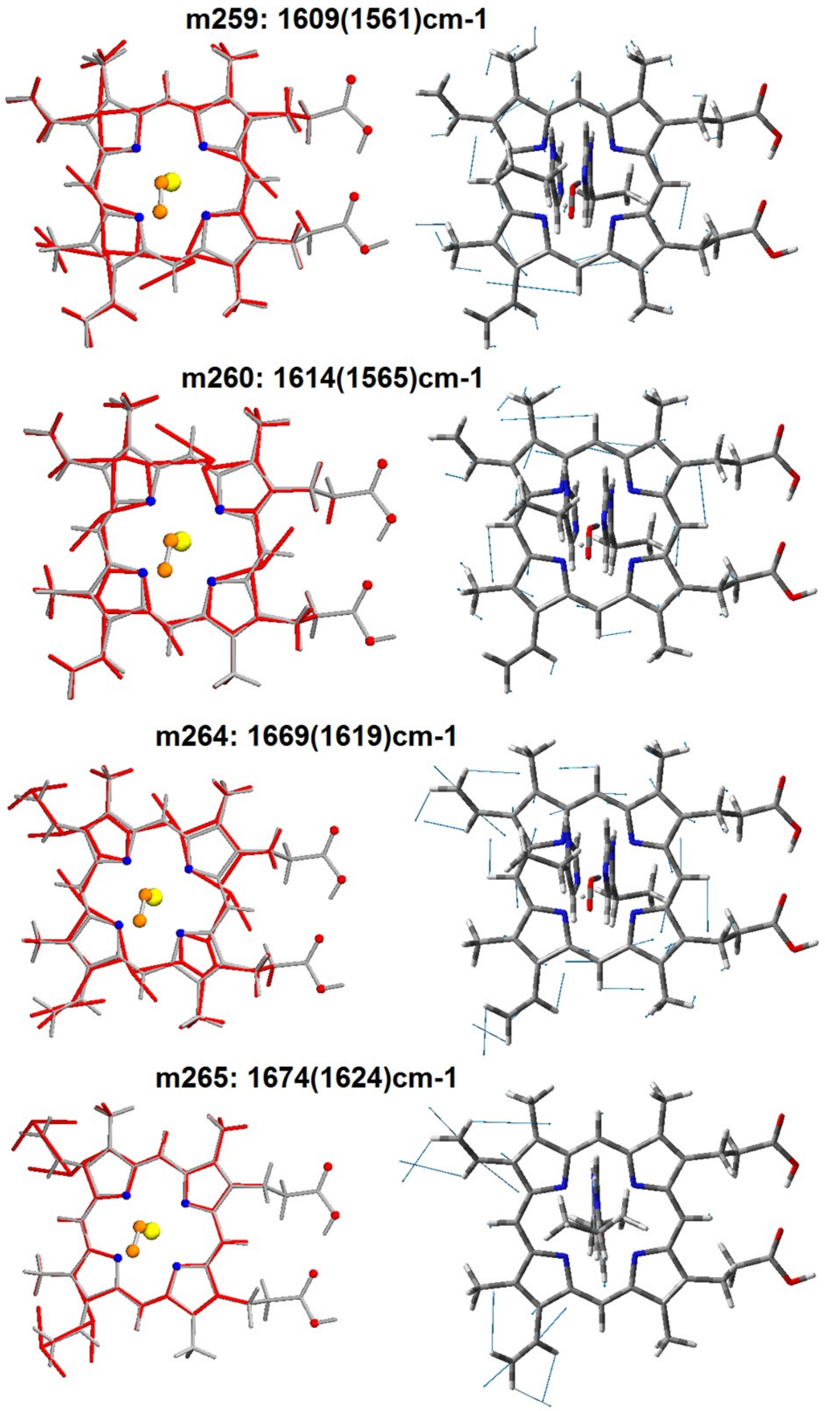

Fig. S19.

Graphical presentations of the normal modes (as indicated) that contribute to the resonant Raman of the oxy-Hb biradical. Left**:** structural changes (red lines) along the normal modes about the equilibrium structure (grey line): to help visualization, we remove histidines. Right**:** displacement vectors mapped on the equilibrium structure of the oxy-Hb biradical.

IMAGES OF ATOMIC DISPLACEMENTS OF MAIN NORMAL MODES COMPUTED FOR DEOXY-HEME

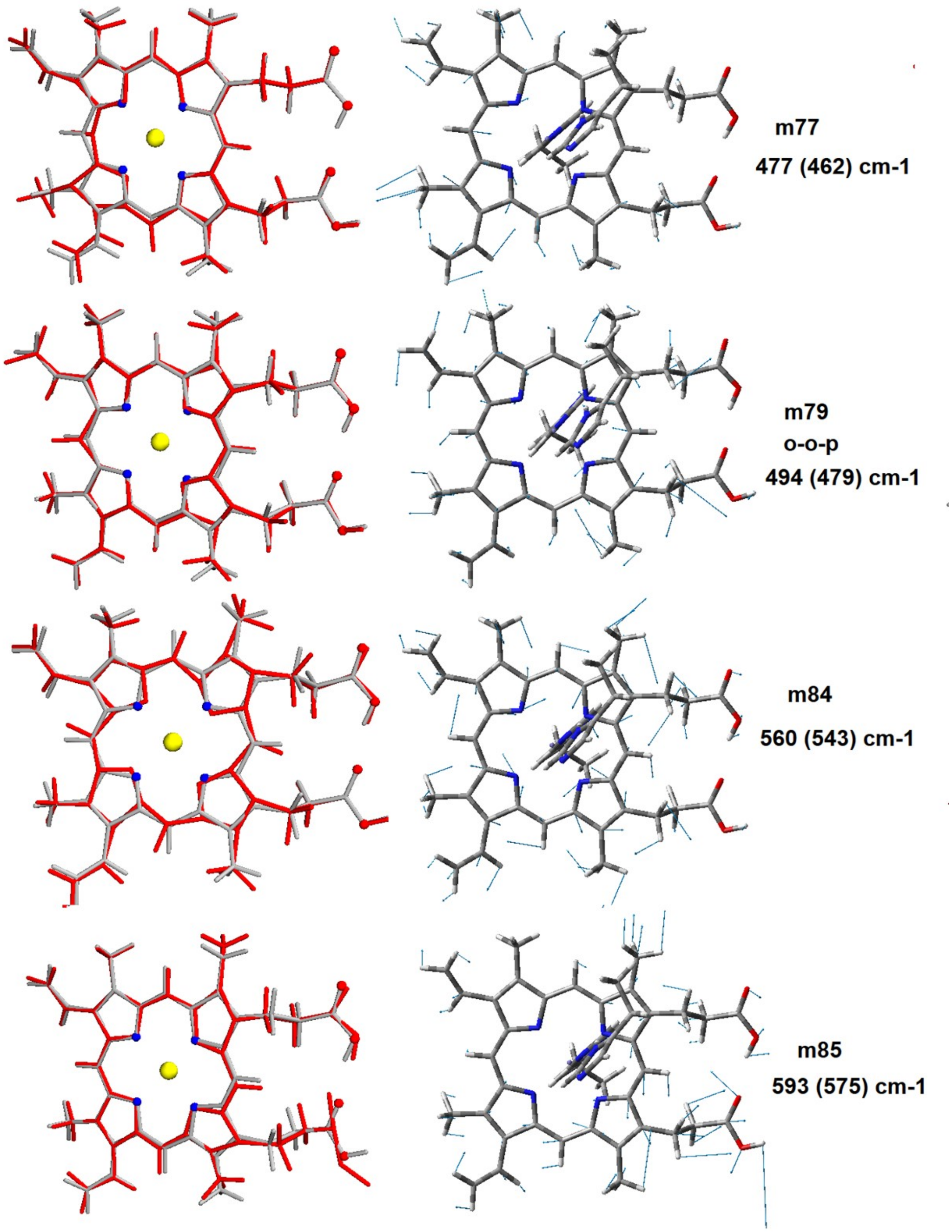

Fig. S20.

Graphical presentations of the normal modes (as indicated) that contribute to the resonant Raman of deoxy-Hb. Left**:** structural changes (red lines) along the normal modes about the equilibrium structure (grey line): to help visualization, we remove histidines. Right**:** displacement vectors mapped on the equilibrium structure of deoxy-Hb.

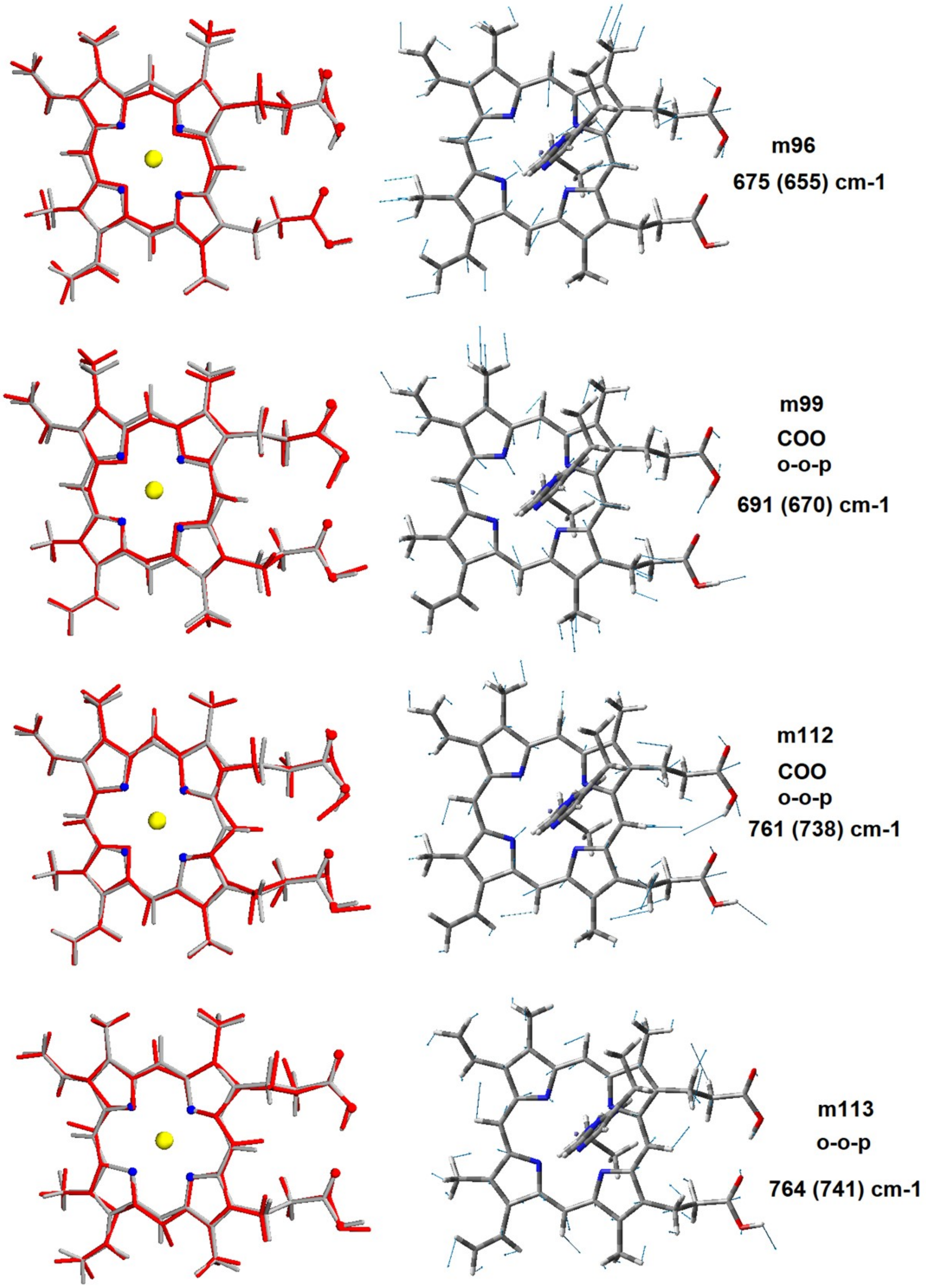

Fig. S21.

Graphical presentations of the normal modes (as indicated) that contribute to the Resonant Raman of deoxy-Hb. Left**:** structural changes (red lines) along the normal modes about the equilibrium structure (grey line): to help visualization, we remove histidines. Right**:** displacement vectors mapped on the equilibrium structure of deoxy-Hb.

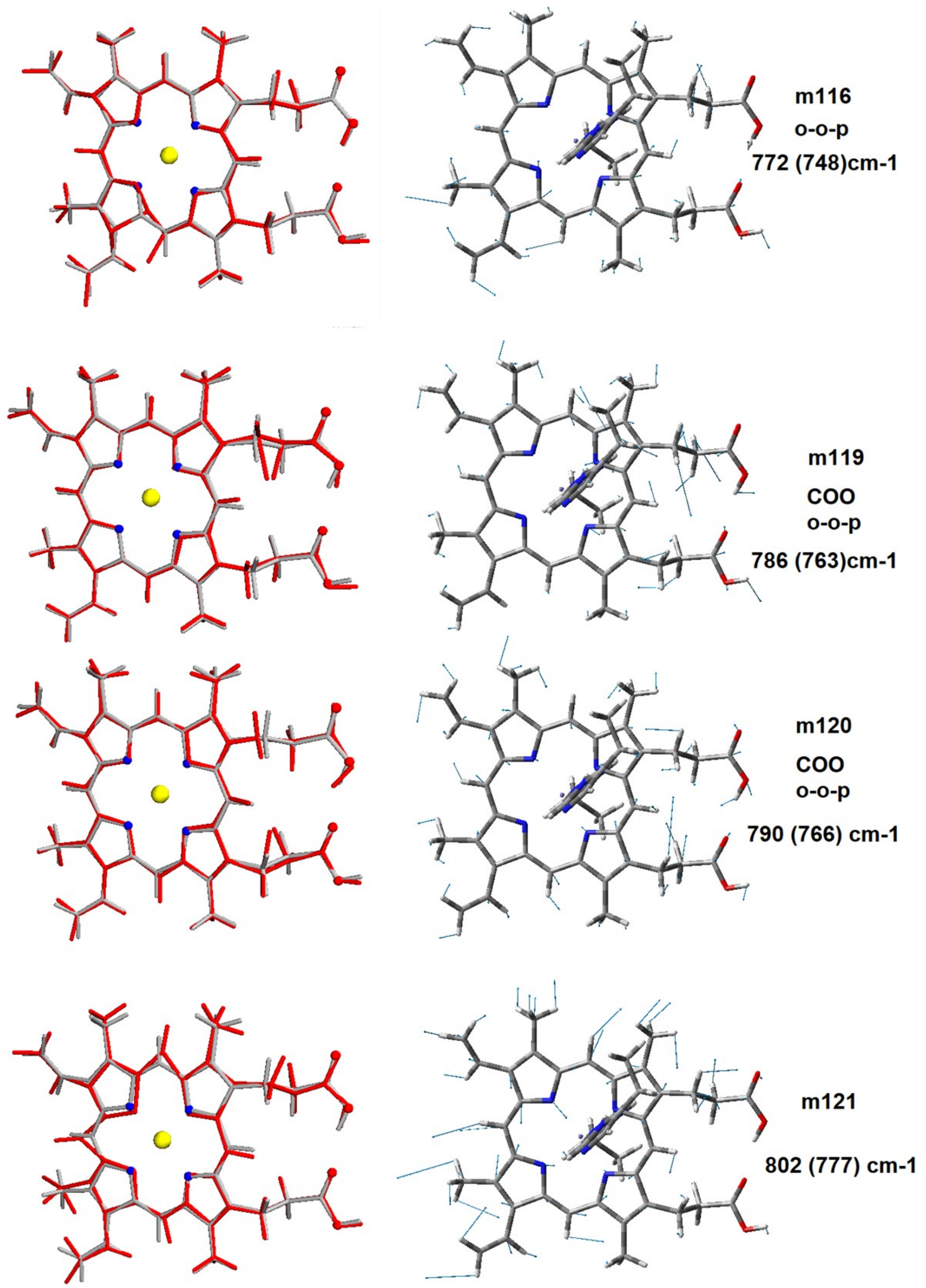

Fig. S22.

Graphical presentations of the normal modes (as indicated) that contribute to the Resonant Raman of deoxy-Hb. Left**:** structural changes (red lines) along the normal modes about the equilibrium structure (grey line): to help visualization, we remove histidines. Right**:** displacement vectors mapped on the equilibrium structure of deoxy-Hb.

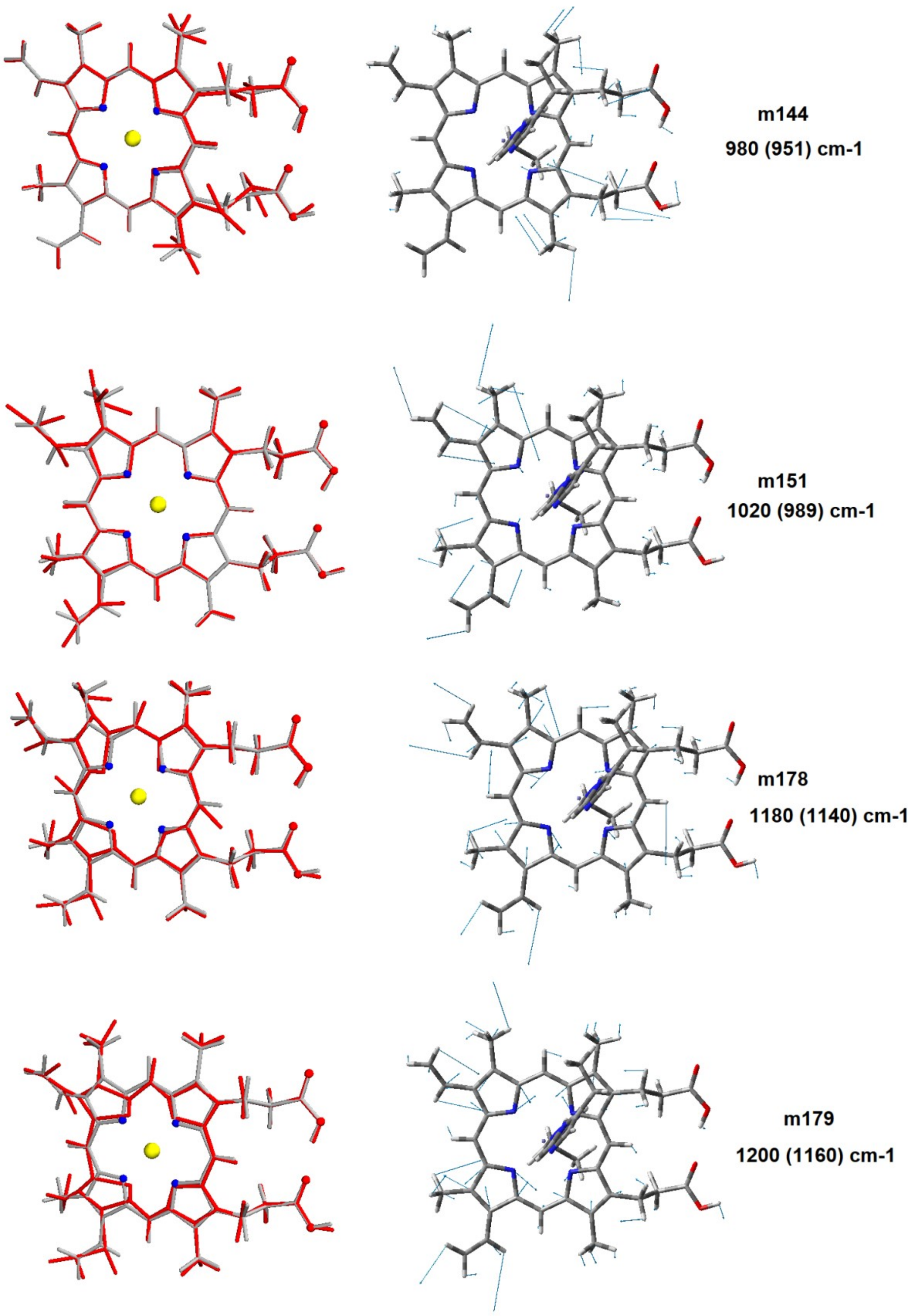

Fig. S23.

Graphical presentations of the normal modes (as indicated) that contribute to the Resonant Raman of deoxy-Hb. Left**:** structural changes (red lines) along the normal modes about the equilibrium structure (grey line): to help visualization, we remove histidines. Right**:** displacement vectors mapped on the equilibrium structure of deoxy-Hb.

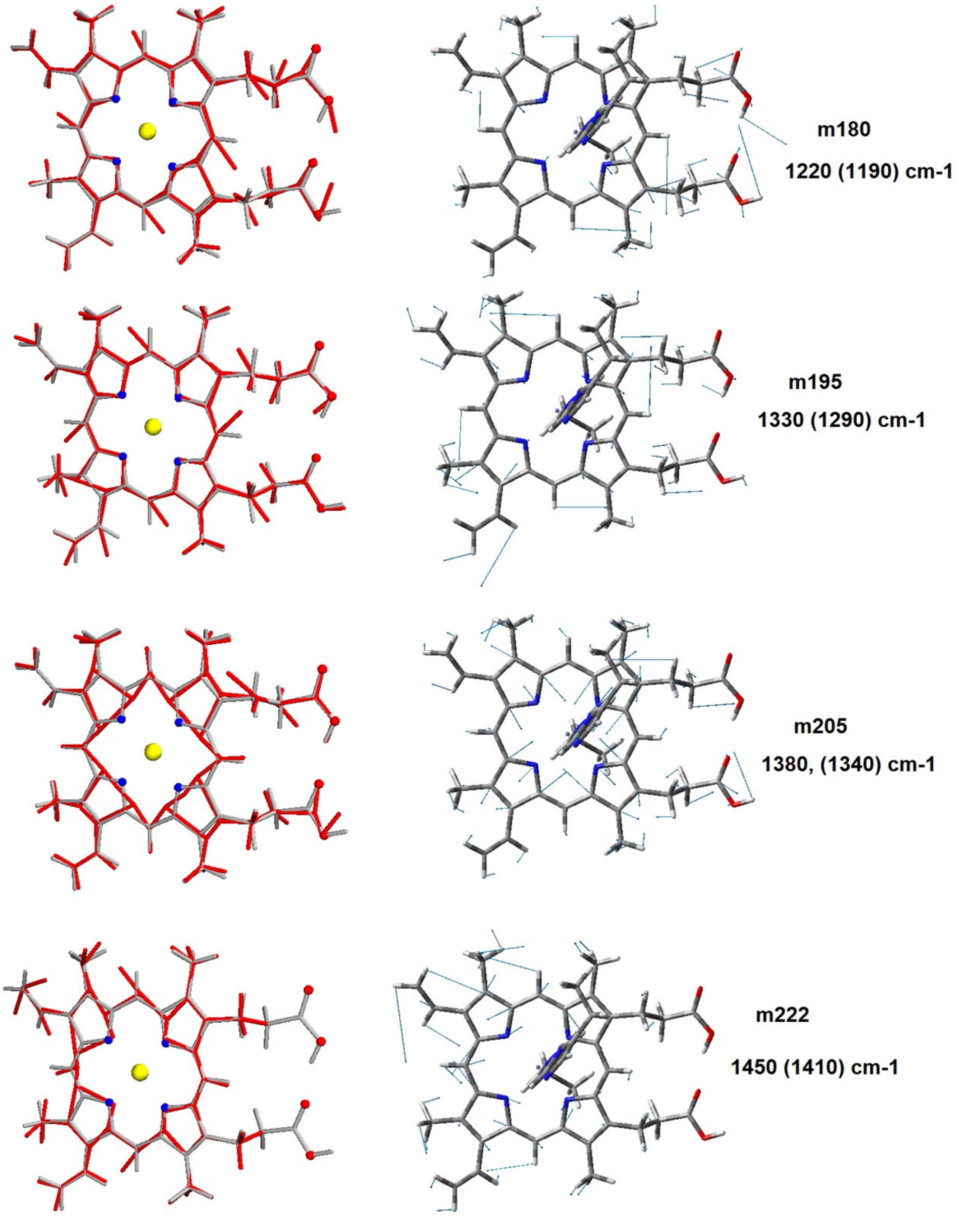

Fig. S24.

Graphical presentations of the normal modes (as indicated) that contribute to the Resonant Raman of deoxy-Hb. Left**:** structural changes (red lines) along the normal modes about the equilibrium structure (grey line): to help visualization, we remove histidines. Right**:** displacement vectors mapped on the equilibrium structure of deoxy-Hb.

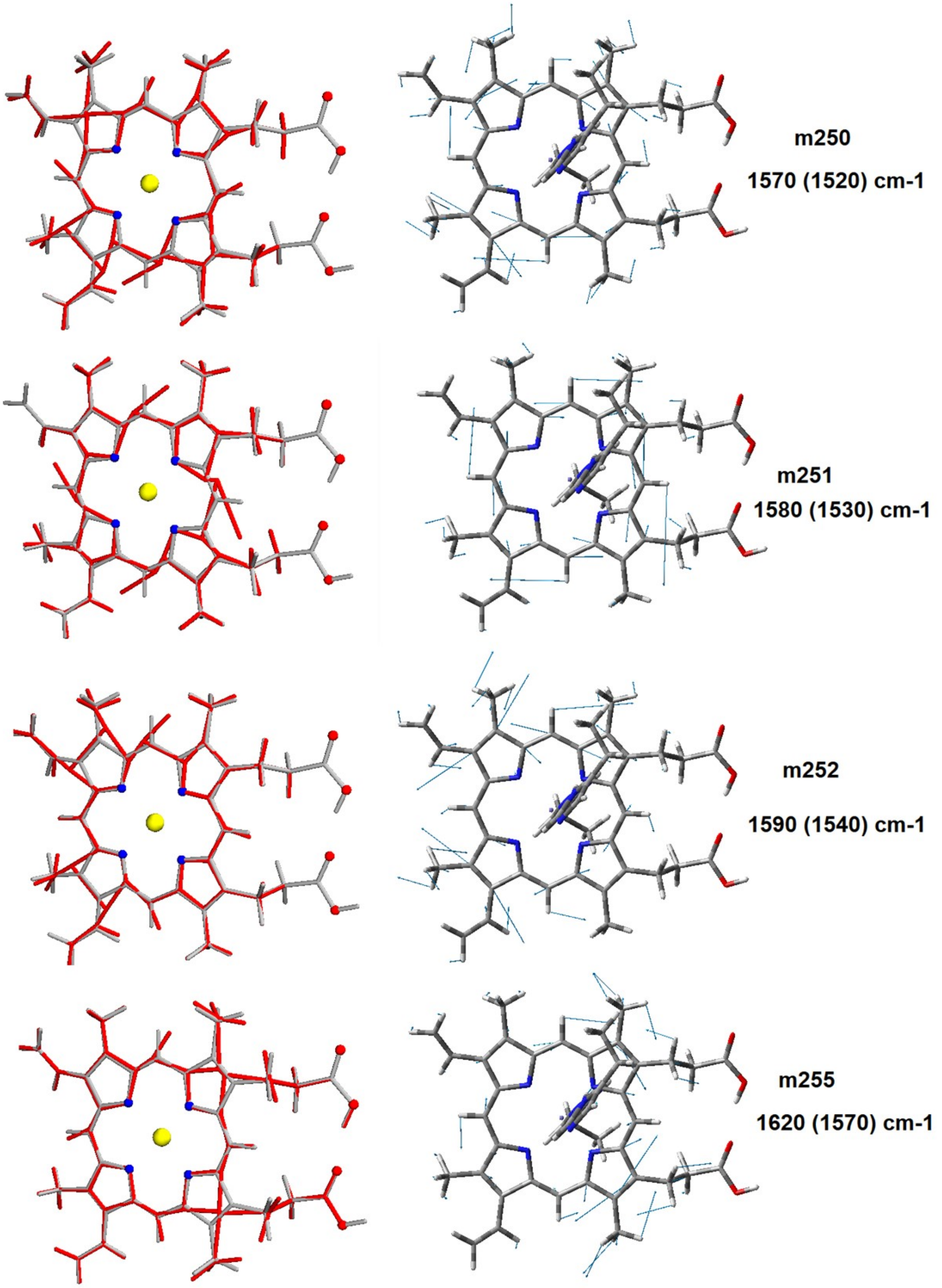

Fig. S25.

Graphical presentations of the normal modes (as indicated) that contribute to the Resonant Raman of deoxy-Hb. Left: structural changes (red lines) along the normal modes about the equilibrium structure (grey line): to help visualization, we remove histidines. Right**:** displacement vectors mapped on the equilibrium structure of deoxy-Hb.

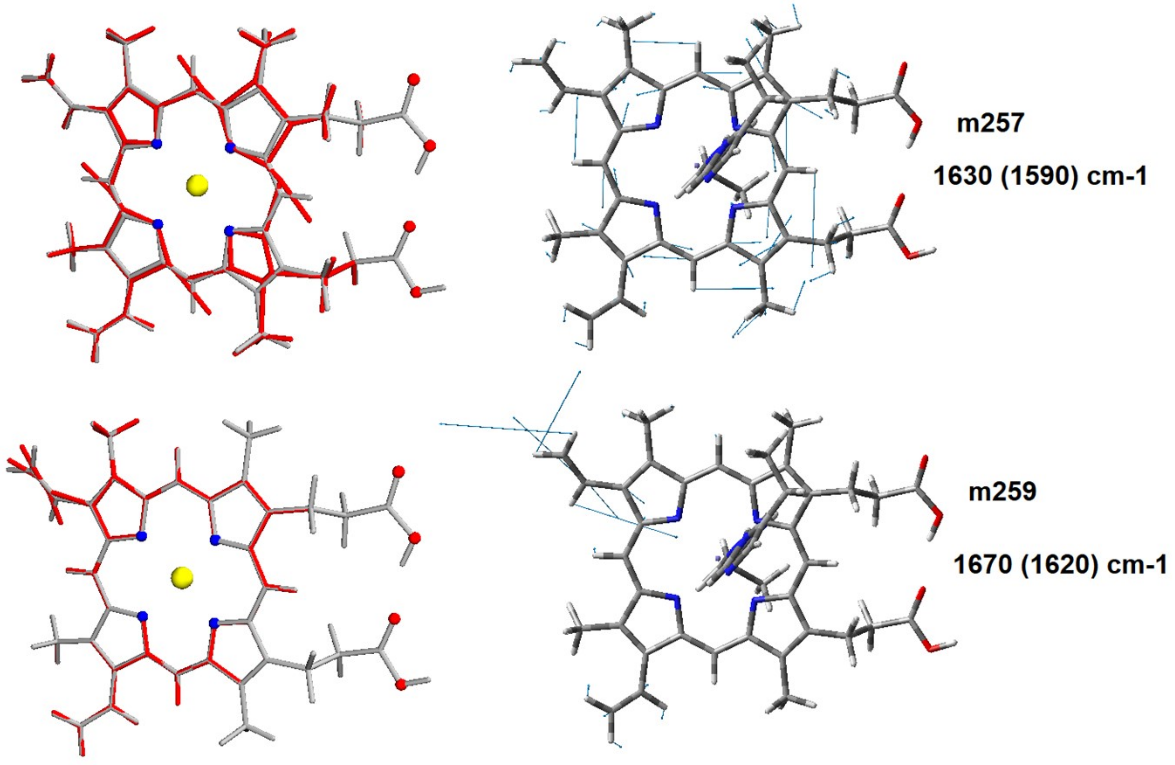

Fig. S26.

Graphical presentations of the normal modes (as indicated) that contribute to the resonant Raman of deoxy-Hb. Left**:** structural changes (red lines) along the normal modes about the equilibrium structure (grey line): to help visualization, we remove histidines. Right**:** displacement vectors mapped on the equilibrium structure of deoxy-Hb.

IMAGES OF ATOMIC DISPLACEMENTS OF MAIN NORMAL MODES COMPUTED FOR MET-HEME

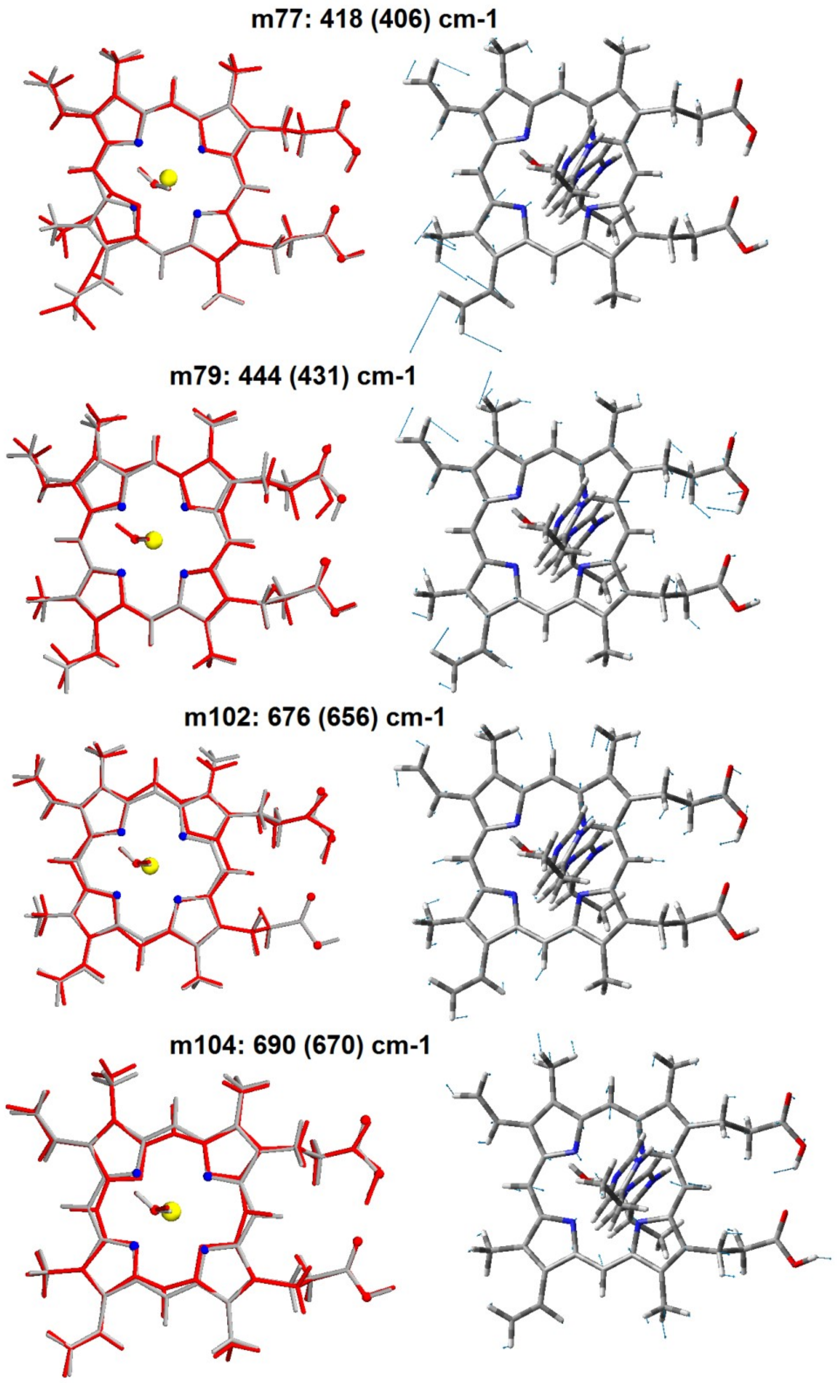

Fig. S27.

Graphical presentations of the normal modes (as indicated) that contribute to the resonant Raman of met-Hb. Left: structural changes (red lines) along the normal modes about the equilibrium structure (grey line): to help visualization, we remove histidines. Right**:** displacement vectors mapped on the equilibrium structure of met-Hb.

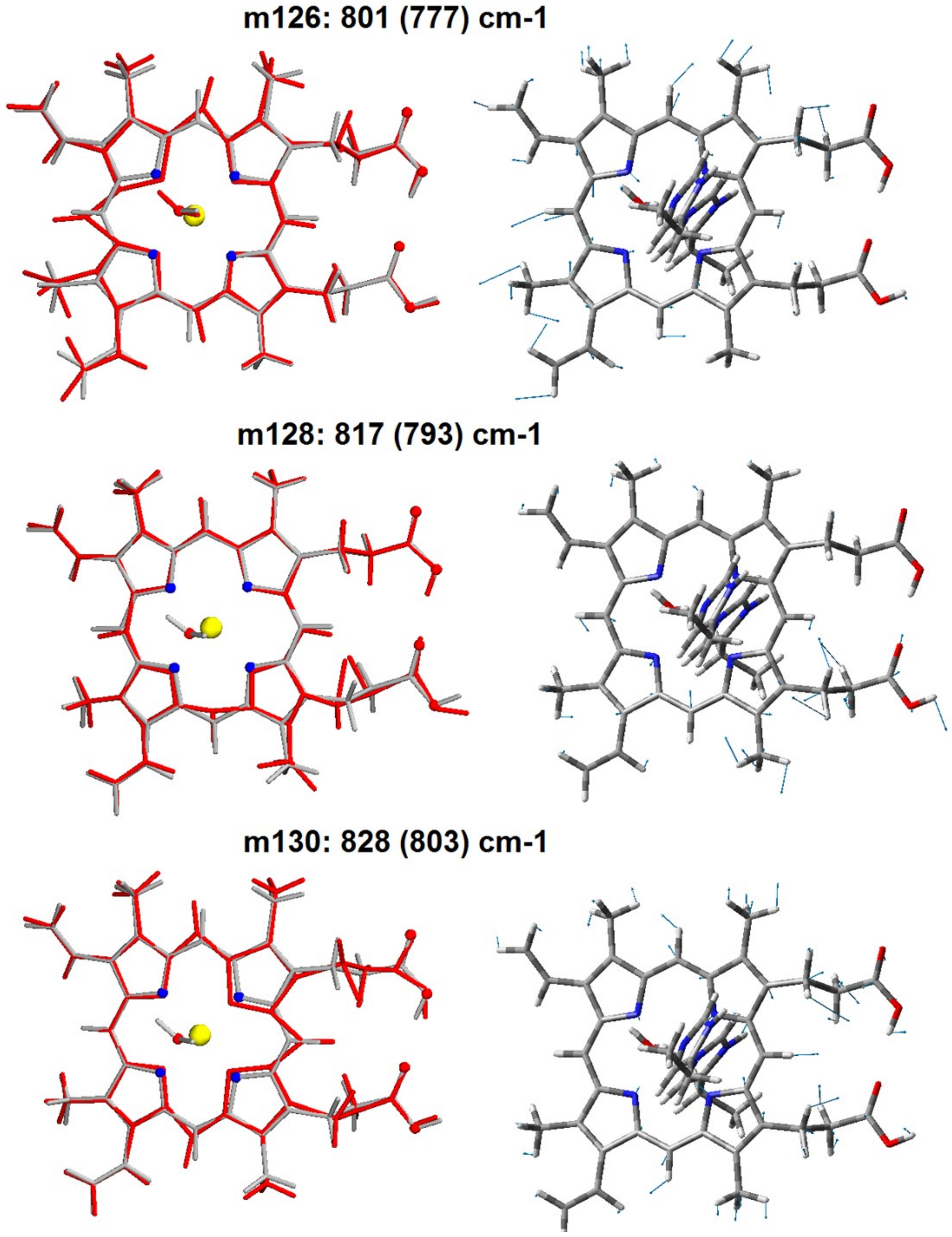

Fig. S28.

Graphical presentations of the normal modes (as indicated) that contribute to the resonant Raman of met-Hb. Left: structural changes (red lines) along the normal modes about the equilibrium structure (grey line): to help the left visualizations, we removed histidines. Right**:** displacement vectors mapped on the equilibrium structure of met-Hb.

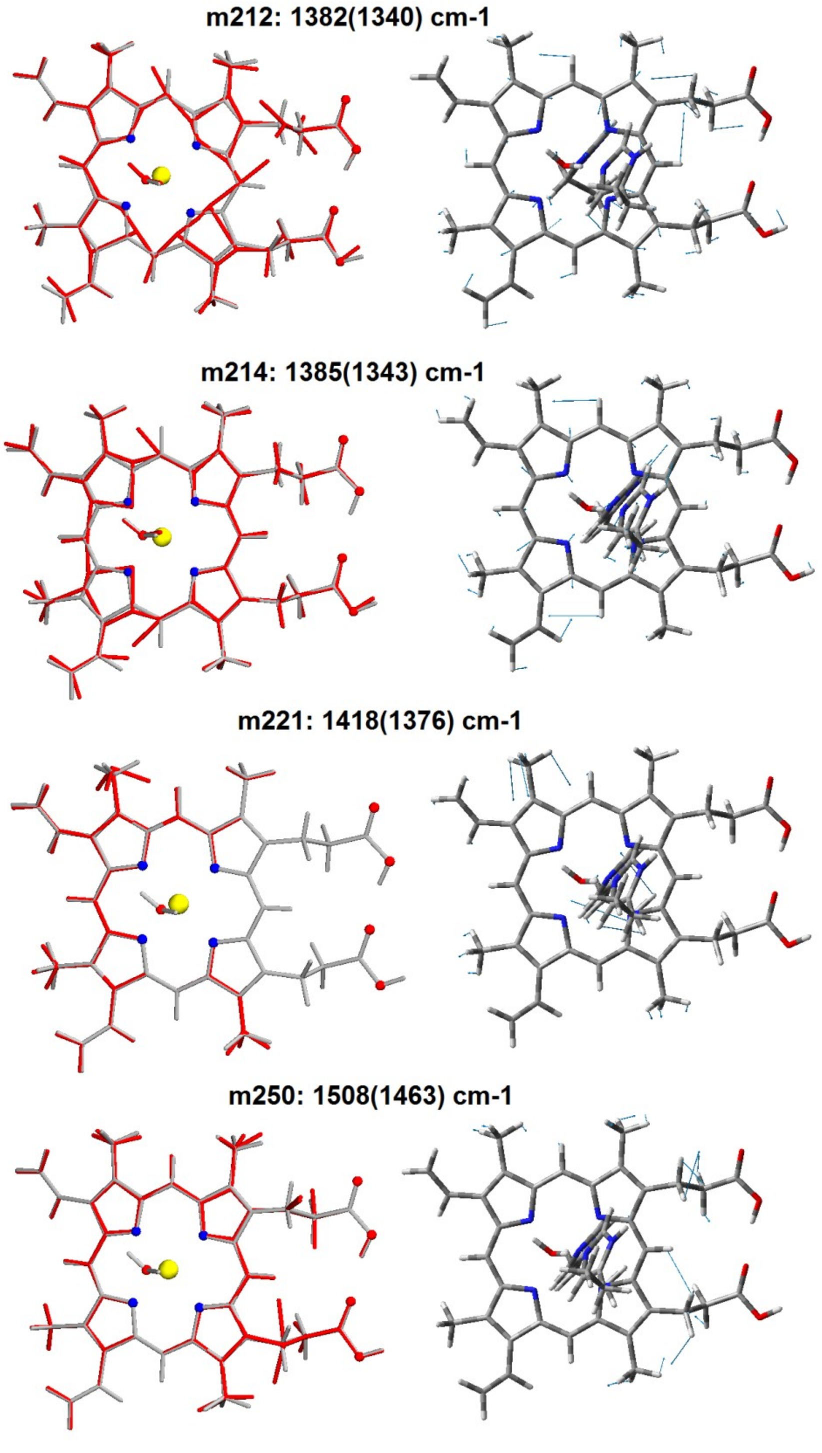

Fig. S29.

Graphical presentations of the normal modes (as indicated) that contribute to the resonant Raman of met-Hb. Left: structural changes (red lines) along the normal modes about the equilibrium structure (grey line): to help the left visualizations, we removed histidines. Right**:** displacement vectors mapped on the equilibrium structure of met-Hb.

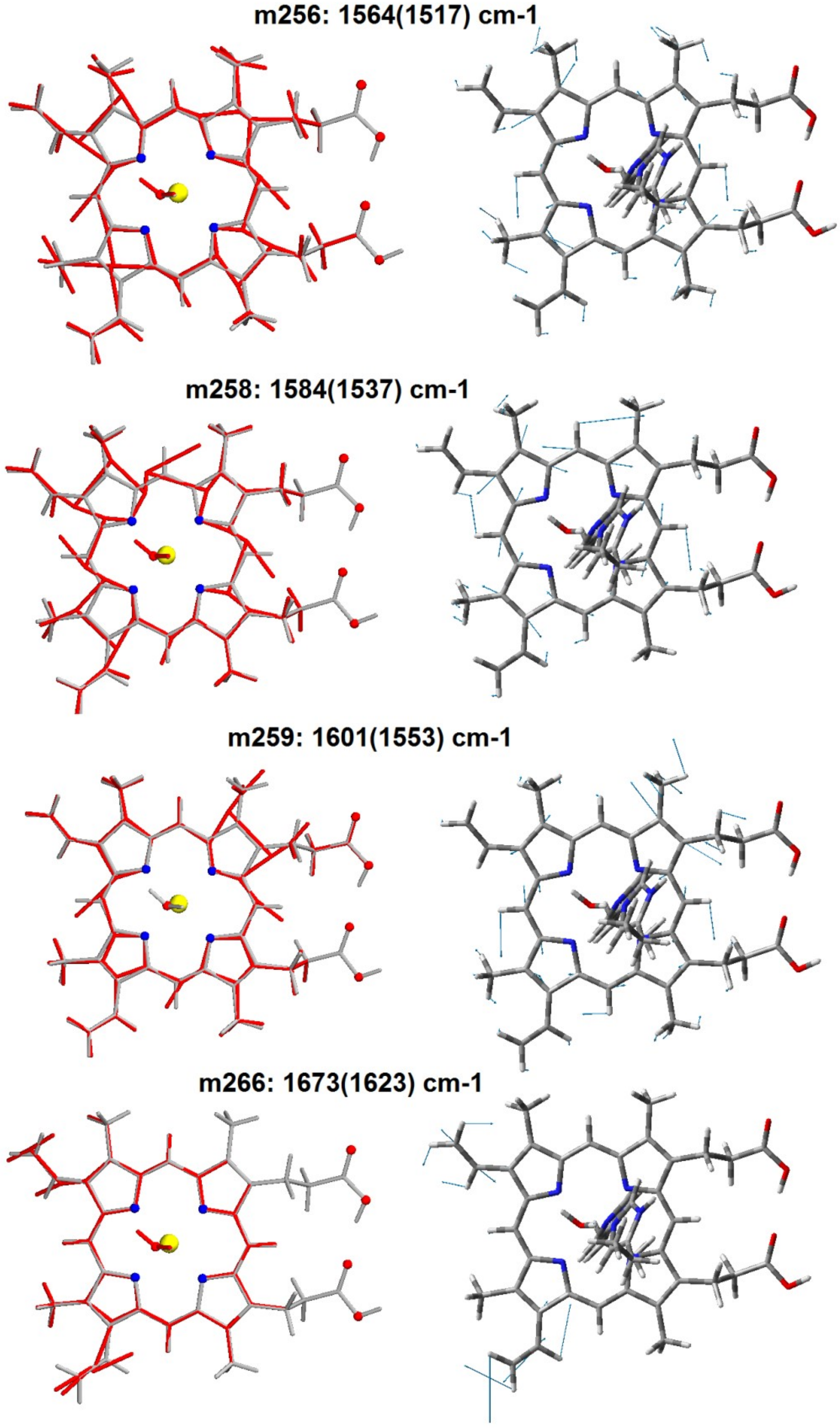

Fig. S30.

Graphical presentations of the normal modes (as indicated) that contribute to the resonant Raman of met-Hb. Left: structural changes (red lines) along the normal modes about the equilibrium structure (grey line): to help the left visualizations, we removed histidines. Right**:** displacement vectors mapped on the equilibrium structure of met-Hb.

RAMAN MICROSCOPY STUDIES

Motivated and instructed by the outcome of the comparative (experimental and theoretical) studies of resonant Raman responses of different forms of hemoglobin in solution and of model heme systems, here we approach the sampling of biochemistry *in vivo* under microscopy resolution. To do this, first, in Fig. S31 we select such spectra sampled in different spatial regions of single discocyte and echinocyte cells such that comparatively the differences would help to "contrast" resonances which we suggest to be specific to met, oxy and deoxy forms of hemoglobin according to the results of the preceding studies.

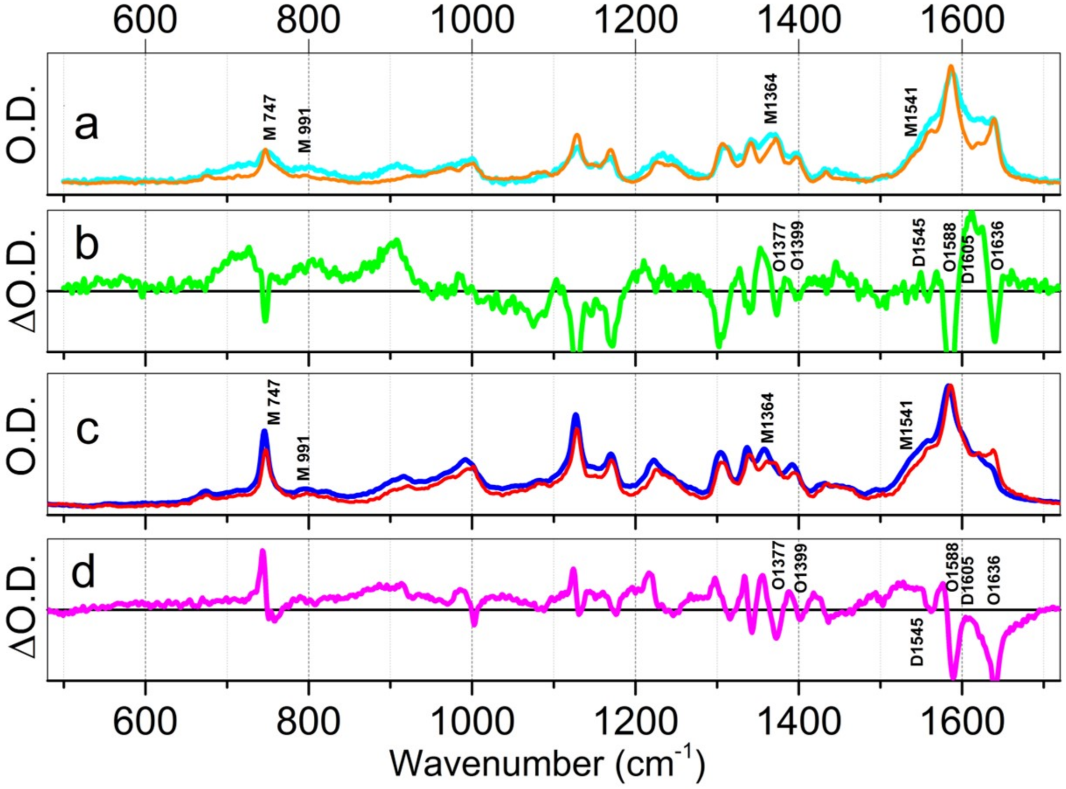

Fig. S31.

Raman spectra of single red blood cells. Representative spectra sampled at different sites of discocyte (**A**) and their differences (**B**). Representative spectra sampled at different sites of echinocyte (**C**) and their differences (**D**).

Accordingly, in Fig. S32, S33 and S34 we plot the differences of Raman maps reconstructed using Raman scattering intensities to explore how consistent (or not) the patterns would express for different resonances under the initial assignment.

Here it is important to stress that we reconstruct images at resonances of interests *without* assistance of linear algebra eigen-component data sorting. In our studies we take Raman scattering at the resonances of interests as detected. At the same time we tested extraction of intensities using deconvolution. Due to the relatively high efficiency and adequate spectral resolution (down to 1 cm^{-1}) of the detected resonant Raman responses from red blood cells, both approaches yield analogous results.

In Fig. S32 we compare Raman difference maps (we subtract images reconstructed for resonances considered specific to oxy-Hb from such of deoxy-Hb) using specific resonances as indicated in the caption. There is a general consistency, which, however, is expected to be possibly compromised since we sample from living dynamic cells where different complexities are expected. In particular, it is reported that under oxidative stress conditions, different forms of hemoglobin would associate with membrane Band 3 complex with different efficiency and the conversion rate between the main forms of hemoglobin would depend on how proximal they are distributed next to the membrane (*S15,S16*). Hence we may anticipate possible anisotropy in distributions of different forms of hemoglobin in dependence on 1) where we would sample the signal, 2) what would be the local shape of the membrane envelope, 3) how stressful or pathologic a selected cell would "feel". In this respect, mathematical modelling of possible hidden anisotropy in typical red blood cell envelopes as we conducted recently (*S17*) is promising and very possible since in the current study we extract Raman tensors.

In the main text of the article we develop a discussion to articulate certain aspects of red blood cell biochemistry. Here we would like to notice that exploring live dynamics in patterns of Raman difference maps to correlate with external environment (using microfluidic devices), in correlation to fluorescence microscopy imaging of actin networks may become a paradigm approach for fast patient blood responses at the level of cellular biochemistry what would allow a better intelligence in intensive care when fast but quality interventions are often required.

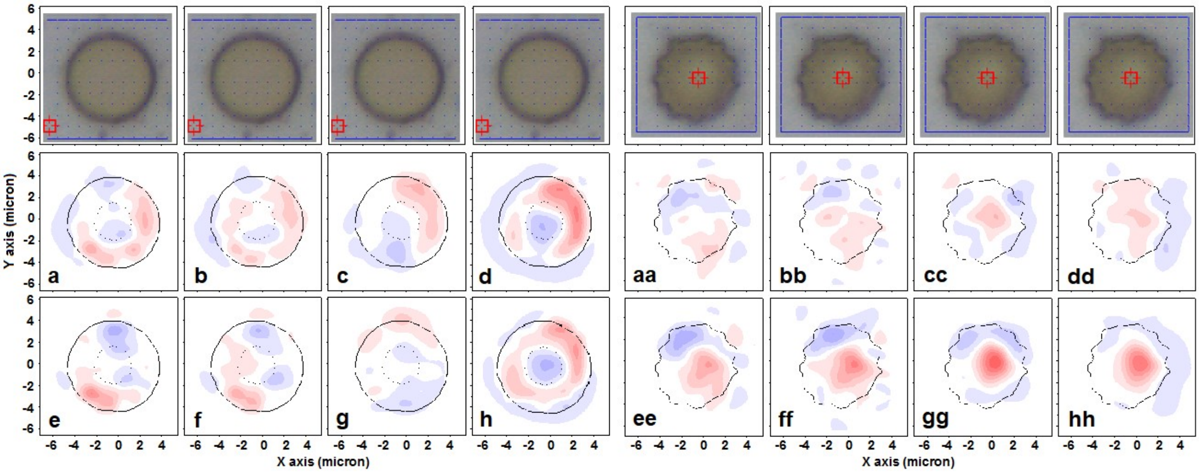

Fig. S32.

Raman microscopy studies. Top: replicas of bright field image of a healthy discocyte (left 4 images) and of a leaky echinocyte (right 4 images) for eye guide to review Raman difference images (below). (**A – D**), differences of Raman images IM_{DR1}-IM_{OR1}, IM_{DR1}-IM_{OR2}, IM_{DR1}-IM_{OR3}, IM_{DR1}- IM_{OR4}, respectively, specific to the selected discocyte. (**E – H**), IM_{DR2}-IM_{OR1}, IM_{DR2}-IM_{OR2}, IM_{DR2}-IM_{OR3}, IM_{DR2}-IM_{OR4}, respectively, specific to the discocyte. Here, IM_{DR1} and IM_{DR2} indicate contributions of images reconstructed using Raman at 1545 and 1605 cm^{-1}, respectively, which we adopt as signatures of deoxy-Hb. Similarly, IM_{OR1}, IM_{OR2}, IM_{OR3} and IM_{OR4} indicate contributions of images reconstructed using Raman scattering at 1377, 1399, 1588 and 1636 cm^{-1}, respectively, which we adopt as signatures of oxy-Hb. Panels (**AA –HH**) present the same content as in panels (**A – H**), but for the selected echinocyte.

Here, DR1 and DR2 indicate that IM_{DR1} and IM_{DR2} images were reconstructed using Raman scattering at 1545 and 1605 cm^{-1}, respectively, which we adopt as signatures of deoxy-Hb. Consistently IM_{OR1}, IM_{OR2}, IM_{OR3} and IM_{OR4} indicate images reconstructed using Raman scattering at 1377, 1399, 1588 and 1636 cm^{-1}, respectively, which we adopt as signatures of oxy-Hb. Panels (**EE – HH**) present the same content as in panels (**E – H**)**,** but for the selected echinocyte. The black contour lines added to the images are to represent the silhouettes of the cells we extract using images at the top of the plate.

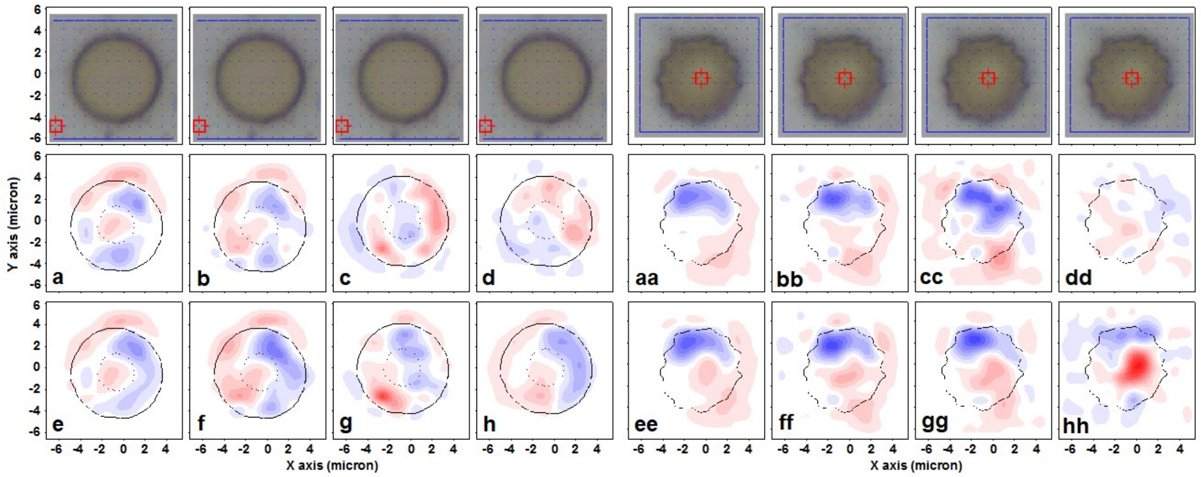

Fig. S33.

Raman microscopy studies. Top: replicas of bright field image of a healthy discocyte (left 4 images) and of a leaky echinocyte (right 4 images) for eye guide to review Raman difference images (below). (**A – D**) differences of Raman images IM_{DR1}-IM_{MR1}, IM_{DR1}-IM_{MR2}, IM_{DR1}-IM_{MR3}, IM_{DR1}-IM_{MR4}, respectively, specific to the selected discocyte. (**E – H**) IM_{DR2}-IM_{MR1}, IM_{DR2}-IM_{MR2}, IM_{DR2}-IM_{MR3}, IM_{DR2}-IM_{MR4}, respectively, specific to the discocyte. Here, DR1 and DR2 as defined in the caption to Fig. S32. Here, IM_{DR1} and IM_{DR2} indicate contributions of images reconstructed using Raman at 1545 and 1605 cm^{-1}, respectively, which we adopt as signatures of deoxy-Hb. Similarly, IM_{MR1}, IM_{MR2}, IM_{MR3} and IM_{MR4} indicate contributions of images reconstructed using Raman scattering at 747, 991, 1364 and 1541 cm^{-1}, respectively, which we adopt as signatures of met-Hb. Panels (**AA – HH**) present the same content as in panels (**A – H**)**,** but for the selected echinocyte.

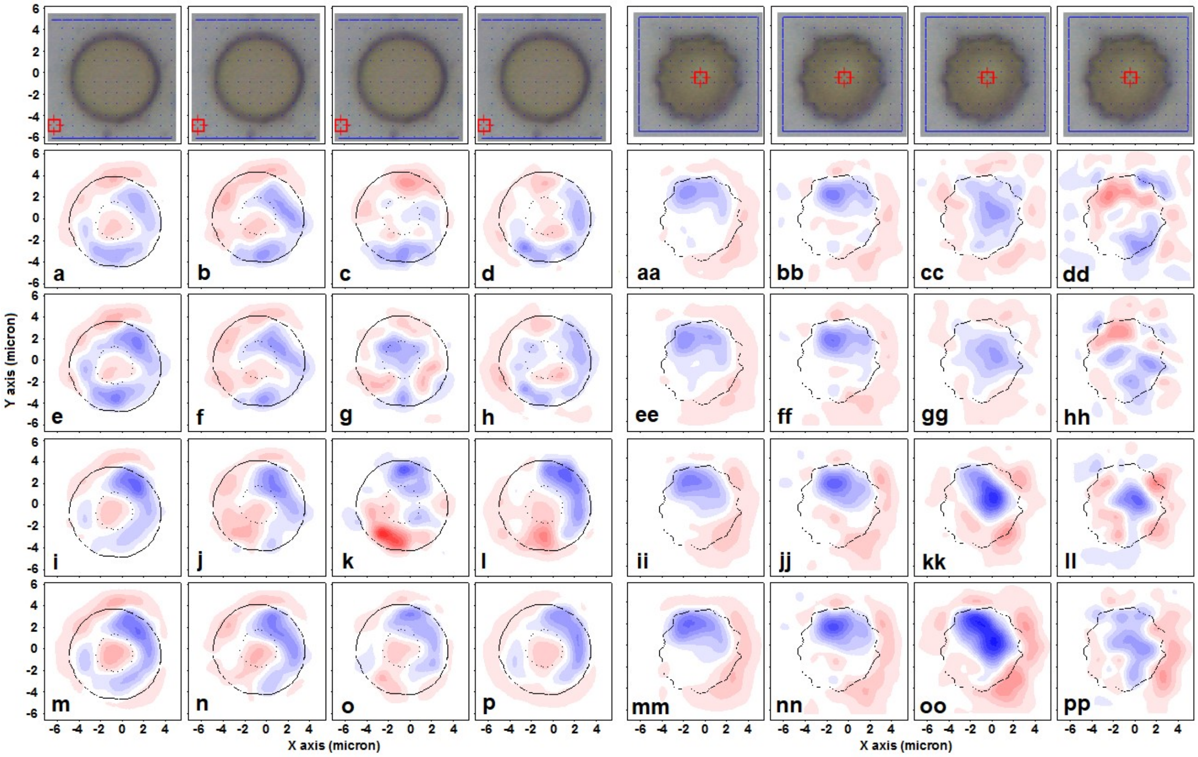

Fig. S34.

Raman microscopy studies. (**A – D**) differences of Raman images IM_{OR1}-IM_{MR1}, IM_{OR1}-IM_{MR2}, IM_{OR1}-IM_{MR3}, IM_{OR1}- IM_{MR4}, respectively, specific to the selected discocyte. (**E – H**) differences of Raman images IM_{OR2}-IM_{MR1}, IM_{OR2}-IM_{MR2}, IM_{OR2}-IM_{MR3}, IM_{OR2}- IM_{MR4}, respectively, specific to the selected discocyte. (**I – L**) differences of Raman images IM_{OR3}-IM_{MR1}, IM_{OR3}-IM_{MR2}, IM_{OR3}-IM_{MR3}, IM_{OR3}- IM_{MR4}, respectively, specific to the selected discocyte. (**M – P**)**,** differences of Raman images IM_{OR4}-IM_{MR1}, IM_{OR4}-IM_{MR2}, IM_{OR4}-IM_{MR3}, IM_{OR4}- IM_{MR4}, respectively, specific to the selected discocyte. Here, IM_{OR1}, IM_{OR2}, IM_{OR3} and IM_{OR4} indicate contributions of images reconstructed using Raman scattering at 1377, 1399, 1588 and 1636 cm^{-1}, respectively, which we adopt as signatures of oxy-Hb. Similarly, IM_{MR1}, IM_{MR2}, IM_{MR3} and IM_{MR4} indicate contributions of images reconstructed using Raman scattering at 747, 991, 1364 and 1541 cm^{-1}, respectively, which we adopt as signatures of met-Hb. Panels (**AA – PP**) present the same content as in panels (**A – P**)**,** but for the selected echinocyte.

SUPPLEMENTARY REFERENCES

S1. Y. Sugita, M. Nagai, Y. Yoneyama, *J. Bio. Chem.* **246,** 383-388 (1971).

S2. R. L. Martin, *J. Chem. Phys.* **118,** 4775-4777 (2003).

S3. T. H. Dunning, P. J. Hay, in "Modern Theoretical Chemistry" (Plenum, New York, 1977) Vol. 3, pp. 1-28.

S4. M. J. Frisch *et al.*, Gaussian 09 (revision B.01, 2010).

S5. A. D. Becke, *Phys. Rev. A* **38,** 3098-3100 (1988).

S6. W. A. Goddard, B. D. Olafson, *Proc. Natl. Acad. Sci. USA* **72,** 2335-2339 (1975).

S7. E. B. Wilson, J. C. Decous, P. C. Cross, in "Molecular vibrations: the theory of infrared and Raman vibrational spectra" (NewYork: McGraw-Hill, 1955).

S8. G. Fermi, M. F. Perutz, B. Shaanan, R. Fourme, *J. Mol. Biol*. **175**, 159-174 (1984).

S9. M. Paoli *et al.*, *J. Mol. Biol.* **256,** 775 (1996).

S10. G. Zheng, M. Schaefer, M. Karplus, *Biochem.* **52,** 8539-8555 (2013).

S11. S. Shaik, H. Chen, *J. Biol. Inorg. Chem.* **16**, 841-855 (2011).

S12. E. L. Mertz, L. I. Krishtalik, *Proc. Natl. Acad. Sci. USA* **97,** 2081-2086 (2000).

S13. T. Simonson, D. Perahia, *Proc. Natl. Acad. Sci. USA* **92,** 1082-1086 (1995).

S14. C. Amovilli *et al.*, *Adv. Quantum Chem.* **32,** 227-261 (1998).

S15. J. M. Rifkind, Z. Zhang, Z., A. Levy, P. T. Manoharan, *Free Radic. Res. Commun.* **12-13,** 645-652 (1991).

S16. F. M. Low, M. B. Hampton, C. C. Winterbourn, *Antioxid. Redox. Signal.* **10,** 1621-1630 (2008).

S17. V. Volkov, C. C. Perry, *Microsc. Microanal.* **22,** 1128-1145 (2016).
